# Health Care Resource Use and Costs of Rivaroxaban Versus Warfarin Among Nonvalvular Atrial Fibrillation Polypharmacy Patients With Obesity

**DOI:** 10.1161/JAHA.124.036401

**Published:** 2025-01-10

**Authors:** Mark Alberts, Jinghua He, Akshay Kharat, Christopher D. Pericone, Veronica Ashton

**Affiliations:** ^1^ Hartford HealthCare Hartford CT USA; ^2^ Janssen Scientific Affairs LLC, a Johnson & Johnson company Titusville NJ USA; ^3^ Janssen Research & Development LLC, a Johnson & Johnson company Raritan NJ USA

**Keywords:** nonvalvular atrial fibrillation, obesity, polypharmacy, real‐world evidence, rivaroxaban, warfarin, Atrial Fibrillation

## Abstract

**Background:**

The economic burden of nonvalvular atrial fibrillation (NVAF) is substantial. Many patients with NVAF are obese and manage other health conditions requiring multiple medications. This real‐world study compared health care resource use (HRU) and costs for rivaroxaban and warfarin in patients with NVAF who had polypharmacy and obesity.

**Methods and Results:**

We used health care claims databases (Merative MarketScan commercial and Medicare supplemental claims) to identify patients initiating the direct oral anticoagulant rivaroxaban or warfarin with ≥1 diagnostic claim for atrial fibrillation, presence of polypharmacy (based on 3 categories for the number of concurrent medications: 1–4, 5–9, ≥10), and obesity. Cohorts were balanced for demographic and baseline characteristics using propensity score matching. All‐cause and NVAF‐related HRU rates and costs were compared between treatments using rate ratios and adjusted mean differences per patient per year. Eligible patients totaled 95 875, with 19 990 patients in each treatment cohort following propensity score matching. All‐cause HRU rates were significantly lower with rivaroxaban versus warfarin, and hospital stays were reduced by 3.1 days with rivaroxaban. Mean (95% CI) all‐cause total medical and total health care costs per patient per year were significantly reduced with rivaroxaban versus warfarin (−$4499 [−$5660 to −$3305] and –$1627 [−$2790 to −$438], respectively). NVAF‐related HRU was reduced with rivaroxaban versus warfarin, but total NVAF‐related medical costs were not significantly different between treatment groups ($144 [−$756 to $1079] per patient per year). Subgroup and sensitivity analysis results were generally consistent with the main analysis.

**Conclusions:**

Among patients with NVAF, polypharmacy, and obesity, rivaroxaban was associated with a reduction in HRU and all‐cause costs compared with warfarin.

**Nonstandard Abbreviations and Acronyms**
DOAC: direct oral anticoagulant
HRU: health care resource use
NVAF: nonvalvular atrial fibrillation
PPPY: per patient per year

**Clinical Perspective**

**What Is New?**

- Using US claims data from patients with nonvalvular atrial fibrillation, obesity, and polypharmacy, lower rates of nonvalvular atrial fibrillation–related health care resource use were observed with rivaroxaban versus warfarin, with significant differences in length of hospital stay, outpatient visits, and physician office visits but not emergency department visits.
- Overall all‐cause total health care costs and total medical costs were significantly lower for patients with nonvalvular atrial fibrillation, obesity, and polypharmacy who received rivaroxaban compared with warfarin, with reductions of $1627 and $4499 per patient per year, respectively.

**What Are the Clinical Implications?**

- This analysis demonstrates that patients with nonvalvular atrial fibrillation, obesity, and polypharmacy who received rivaroxaban had lower overall health care resource use and costs compared with patients who received warfarin, which may help inform treatment decisions to ensure the most efficient use of health care resources in this population.

Nonvalvular atrial fibrillation (NVAF) is associated with substantial economic burden, and many patients with NVAF have concurrent medical conditions, including obesity, that require multiple medications. As the prevalence of obesity continues to increase, its association with poor health also leads o substantial medical expenditures.^1^, ^2^, ^3^ Compared with individuals with normal weight, individuals who are overweight and individuals with obesity have increased primary and secondary health care resource use (HRU).^4^, ^5^ Obesity is associated with increased direct health care costs, as well as lost productivity, disability, and mortality.^3^ Individuals with obesity are at a higher risk of developing comorbidities, including atrial fibrillation and thrombotic events.^6^, ^7^, ^8^

In patients with NVAF, oral anticoagulation therapy (eg, a vitamin K antagonist, like warfarin, or direct oral anticoagulants [DOACs]), is standard of care to prevent thromboembolic events.^9^, ^10^ According to 2023 guidelines from the American College of Cardiology, the American Heart Association, the American College of Clinical Pharmacy, and the Heart Rhythm Society, DOACs are recommended over warfarin in patients who have atrial fibrillation and an increased risk of stroke.^10^ This recommendation is not based on body weight, but a new section for patients with atrial fibrillation and class III obesity (body mass index [BMI] of ≥40 kg/m^2^) indicates that post hoc analyses of major trials of DOACs in atrial fibrillation and large observational studies have shown comparable efficacy and safety compared with warfarin across weight groups, and thus DOACs are preferred over warfarin for stroke‐risk reduction except in patients undergoing bariatric surgery due to limited evidence in this population. Recent studies have evaluated the use of DOACs in patients with morbid obesity (defined as BMI of ≥40 kg/m^2^ or body weight ≥120 kg), of which 2 recent systematic reviews suggest that the benefit–risk profile of DOACs is maintained in these patients.^11^, ^12^ Rivaroxaban, an oral direct factor Xa inhibitor, was approved in November 2011 for the prevention of stroke and systemic embolism in patients with NVAF based on the ROCKET‐AF (Rivaroxaban Once‐Daily Oral Direct Factor Xa Inhibition Compared With Vitamin K Antagonism for Prevention of Stroke and Embolism Trial in Atrial Fibrillation) trial.^13^, ^14^ In both drug development and postmarketing studies, the overall pharmacologic and clinical profile of rivaroxaban has been shown to be consistent in adults with obesity, which supports not needing to adjust the dose of rivaroxaban on the basis of BMI or body weight.^15^ In real‐world studies of patients with NVAF across a spectrum of body weights and BMIs, rivaroxaban was associated with fewer hospitalizations and outpatient visits and lower costs compared with warfarin.^16^, ^17^

The occurrence of comorbid conditions in patients with obesity and NVAF leads to the concurrent use of various medications to treat these conditions, in addition to an oral anticoagulant.^18^, ^19^ Polypharmacy, which is defined as the use of multiple medications, may be associated with adverse clinical outcomes and mortality.^20^, ^21^, ^22^ The definition of polypharmacy varies in the literature, but the most commonly reported definition is ≥5 concurrent medications.^23^ This study evaluated HRU and costs of rivaroxaban compared with warfarin in patients with NVAF, obesity, and polypharmacy. Our hypothesis was that rivaroxaban would be associated with reduced HRU and costs compared with warfarin in this high‐risk patient population.

## METHODS

### Data Availability

The data sharing policy of Janssen Pharmaceutical Companies of Johnson & Johnson is available at https://www.janssen.com/clinical‐trials/transparency. The Merative™ MarketScan^®^ Research Databases were used under license for the current study and are not publicly available. V.A. and J.H. had full access to all the data in the study and take responsibility for its integrity and the data analysis.

This study is based on deidentified data collected from a health care claims database and does not contain any experimental data with human or animal participants; this analysis was deemed exempt from institutional review board oversight, and informed consent was not obtained as per guidance from the Office for Human Research Protections.

### Data Sources

Two large health care claims databases were used: Merative™ MarketScan^®^ Commercial Claims and Encounters and Merative MarketScan Medicare Supplemental. The Commercial Claims and Encounters database consists of approximately 138 million unique deidentified individuals, including active employees, early retirees, COBRA continuers, and their dependents who are insured by employer‐sponsored plans. The database is fully adjudicated for paid medical and pharmacy insurance claims for services, including inpatient admissions, outpatient visits, and outpatient pharmacy. The Medicare Supplemental administrative health claims database consists of active and retired employees and their dependents who are eligible for Medicare benefits from employer‐sponsored supplemental plans. This database provides information on individual‐specific clinical use, cost, and enrollment across inpatient, outpatient, prescription drug, and carve‐out services.

### Study Design

This retrospective cohort study began on December 1, 2010, and cohort identification began on December 1, 2011, based on the approval date of November 2011 for rivaroxaban. Patients were followed up to the earliest event of continuous benefit enrollment end or the study period end on March 1, 2020 (Figure S1).

Patients newly initiating rivaroxaban or warfarin between December 1, 2010, and March 1, 2020, were identified from the database with the following inclusion criteria: ≥1 pharmacy claim for rivaroxaban or warfarin during the identification period (Figure S1), with the first claim defined as the index date; ≥12 months of continuous medical and pharmacy benefit enrollment before and on the index date; ≥1 medical claim with a diagnosis of atrial fibrillation during the 12‐month period before or on the index date (*International Classification of Diseases, Ninth Revision* [*ICD‐9*] code 427.31 and *Tenth Revision* [*ICD‐10*] codes I48.0, I48.1, I48.11, I48.19, I148.2, I148.20, I48.21, I48.9, I48.91, I48.92‐); ≥18 years of age; presence of polypharmacy; and presence of obesity on the cohort index date.

The number of concurrent medications used in addition to rivaroxaban or warfarin (1–4, 5–9, and ≥10) on the index date determined the presence of polypharmacy.^19^ Concurrent use was defined by the date of the prescription, days' supply, and a grace period of 14 days (Figure S2). Generic drug names and their generic product identifier code at an 8‐digit level (or 10‐digit level for fixed‐dose combinations) were used to identify different pharmacy prescriptions.

Obesity categories (BMI: 30–34 kg/m^2^, 35–39 kg/m^2^, and ≥40 kg/m^2^) were determined using a proprietary validated BMI interpolation algorithm that has been described in detail previously.^24^ Each patient's baseline profile was used in the predictive models to determine BMI classifications based on claims data.^24^ Two independent BMI prediction algorithms, one for patients with obesity diagnosis codes and the other for patients without obesity diagnosis codes, were used to impute the BMI categories and the results were pooled together.

Exclusion criteria included the following: a diagnosis code for stroke, systemic embolism, or major bleeding within 30 days before the index date; pharmacy claims for ≥2 oral anticoagulant medications on the index date; ≥1 pharmacy claim for an oral anticoagulant at any time before the index date; evidence of another indication for anticoagulation (ie, acute venous thromboembolism, prophylaxis after hip/knee replacement surgery) during the baseline period; a diagnosis code for mitral stenosis; or a diagnosis or procedure code for a mechanical heart‐valve procedure at any time before the index date (see Data S1 for codes).

### Outcomes

All‐cause and NVAF‐related HRU were reported for the following settings: inpatient hospitalization, including length of stay (days); 30‐day rehospitalization; emergency department (ED) visits; physician office visits; and outpatient service encounters. Total health care costs were evaluated as the sum of total medical costs plus pharmacy costs. Total medical costs included costs for inpatient hospitalization, ED visits, physician office visits, outpatient service encounters (including rehabilitation), and skilled nursing facility/long‐term care. Total health care costs were determined for all‐cause costs, including costs associated with monitoring international normalized ratio (noting that monitoring visits were not identified separately), and for NVAF‐related costs. HRU and costs were intent‐to‐treat, meaning the earliest of health plan disenrollment or latest data availability. HRU and costs were considered to be NVAF‐related for claims with NVAF in the first or second billing position.

### Statistical Analysis

A logistic regression model was used to predict the propensity of receiving rivaroxaban with consideration of the following potential confounders. Demographic variables were evaluated on the index date and included age, sex, geographic region, health plan type, insurance type (commercial or Medicare), and index year. Baseline clinical characteristics were measured during the 12‐month baseline period and included Quan‐Charlson Comorbidity Index^25^; CHA_2_DS_2_‐VASc score^26^; HAS‐BLED score^27^; gastric bypass surgery during the 12‐month baseline period; use of nonoral anticoagulants; atrial fibrillation medications; cardiovascular procedures; cardiovascular‐related medications; cancer diagnosis; risk factors for stroke and bleeding^28^; and baseline HRU and costs (see Data S2 and S3 for related codes). Categories for polypharmacy and BMI were also included as covariates.

Propensity score matching was used to reduce potential bias and create more comparable cohorts based on demographic and baseline characteristics. Rivaroxaban users were matched 1:1 with warfarin users without replacement on the logit of the propensity score using calipers of width equal to 20% of the SD of the logit of the propensity score. Exact match was required for age group, sex, insurance type, and index year.

Demographic and baseline characteristics for each treatment cohort were summarized with descriptive statistics; differences in baseline characteristics were assessed using standardized mean differences, with a difference of <10% considered to be a negligible imbalance. Rates of all‐cause and NVAF‐related HRU and costs were calculated as the number of events or costs incurred over the follow‐up period divided by the patient‐years of observation for per patient per year (PPPY) estimates. HRU was compared between treatment cohorts using rate ratios, and mean cost differences between cohorts were calculated. Length of stay was determined as mean differences in days. All costs were inflated to 2020 US dollars based on the medical care component of Consumer Price Index. For both HRU and costs, nonparametric bootstrap procedures were used to estimate 95% CIs and *P* values.

Comparative analyses were repeated for subgroups based on the number of concurrent medications (1–4, 5–9, ≥10) and obesity subgroups (BMI: 30–34 kg/m^2^, 35–39 kg/m^2^, ≥40 kg/m^2^) using the same covariates. Sensitivity analyses were conducted by index year of 2014 and after, obesity based on the presence of baseline obesity diagnosis codes, and an on‐treatment approach that ended the follow‐up period for index treatment discontinuation or switching/adding other anticoagulants.

## RESULTS

### Patient Characteristics

**Figure 1:**
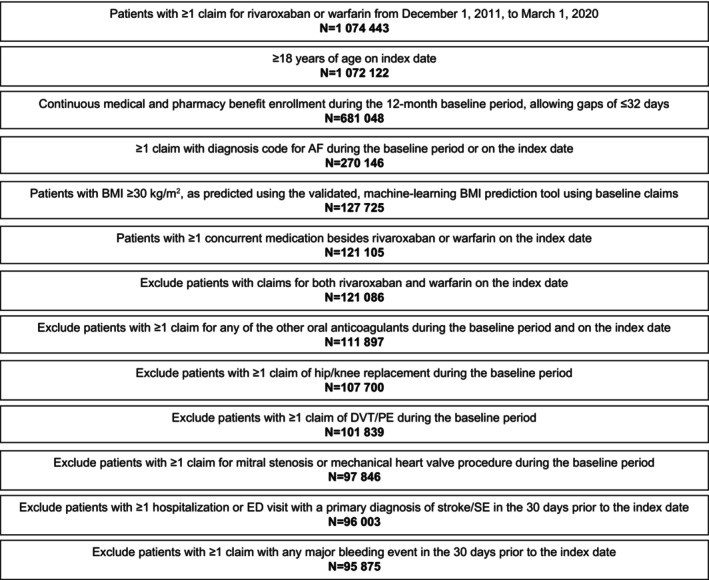
Patient attrition/disposition. AF indicates atrial fibrillation; BMI, body mass index; DVT, deep vein thrombosis; ED, emergency department; PE, pulmonary embolism; and SE systemic embolism.

A total of 95 875 patients met eligibility criteria, with 33 191 having received rivaroxaban and 62 684 having received warfarin (Figure 1). Before matching, patients treated with rivaroxaban were younger; more likely to be receiving 1 to 4 concurrent medications; and had lower Quan‐Charlson Comorbidity Index @, CHA_2_DS_2_‐VASc, and HAS‐BLED scores compared with patients treated with warfarin (Table 1). Baseline HRU and costs were lower in patients receiving rivaroxaban.

**Table 1:** Demographic and Baseline Characteristics Before and After Matching

Characteristic	Before matching			After matching		
	Rivaroxaban (n=33 191)	Warfarin (n=62 684)	Standardized mean difference*	Rivaroxaban (n=19 990)	Warfarin (n=19 990)	Standardized mean difference*
Age, y, median (IQR)	62 (57–70)	68 (61–76)	46.2%	64 (59–73)	64 (59–73)	0.7%
Sex, n (%)						
Male	21 872 (65.9)	39 110 (62.4)	7.3%	12 714 (63.6)	12 714 (63.6)	0.0%
Female	11 319 (34.1)	23 574 (37.6)	7.3%	7276 (36.4)	7276 (36.4)	0.0%
Insurance type, n (%)						
Commercial	21 089 (63.5)	26 326 (42.0)	44.2%	10 749 (53.8)	10 749 (53.8)	0.0%
Medicare	12 102 (36.5)	36 358 (58.0)	44.2%	9241 (46.2)	9241 (46.2)	0.0%
Baseline risk scores, median (IQR)						
Quan‐Charlson Comorbidity Index	1 (0–2)	2 (0–3)	25.5%	1 (0–3)	2 (0–3)	7.7%
CHA_2_DS_2_‐VASc	2 (1–4)	3 (2–5)	42.1%	3 (2–4)	3 (2–4)	5.3%
HAS‐BLED	2 (1–3)	2 (1–3)	18.4%	2 (1–3)	2 (1–3)	3.5%
Baseline concurrent medications, n (%)						
1 to 4	14 585 (43.9)	20 446 (32.6)	23.5%	7961 (39.8)	7901 (39.5)	0.6%
5 to 9	14 130 (42.6)	30 603 (48.8)	12.6%	8942 (44.7)	9010 (45.1)	0.7%
≥10	4476 (13.5)	11 635 (18.6)	13.9%	3087 (15.4)	3079 (15.4)	0.1%
Baseline obesity class, n (%)						
Class I (BMI 30–34 kg/m^2^)	16 340 (49.2)	32 865 (52.4)	6.4%	10 215 (51.1)	10 044 (50.3)	1.7%
Class II (BMI 35–39 kg/m^2^)	5156 (15.5)	9378 (15.0)	1.6%	2926 (14.6)	2894 (14.5)	0.5%
Class III (BMI ≥40 kg/m^2^)	11 695 (35.2)	20 441 (32.6)	5.5%	6849 (34.3)	7052 (35.3)	2.1%
Most common baseline comorbidities (>10%†), n (%)						
Hypertension	28 370 (85.5)	51 146 (81.6)	10.5%	17 002 (85.1)	16 827 (84.2)	2.4%
Hyperlipidemia	21 418 (64.5)	37 960 (60.6)	8.2%	12 849 (64.3)	12 547 (62.8)	3.1%
Diabetes without chronic complications	13 517 (40.7)	31 778 (50.7)	20.1%	8972 (44.9)	9304 (46.5)	3.3%
Congestive heart failure	8953 (27.0)	24 701 (39.4)	26.6%	6354 (31.8)	7055 (35.3)	7.4%
Osteoarthritis	8303 (25.0)	16 382 (26.1)	2.6%	5266 (26.3)	5133 (25.7)	1.5%
Chronic pulmonary disease	8240 (24.8)	18 839 (30.1)	11.7%	5495 (27.5)	5791 (29.0)	3.3%
Cancer	7865 (23.7)	19 306 (30.8)	16.0%	5566 (27.8)	5500 (27.5)	0.7%
Thyroid disease	6395 (19.3)	11 691 (18.7)	1.6%	4031 (20.2)	3763 (18.8)	3.4%
Coronary artery disease	5366 (16.2)	10 179 (16.2)	0.2%	3093 (15.5)	3662 (18.3)	7.6%
Chronic obstructive pulmonary disease	4488 (13.5)	12 173 (19.4)	16.0%	3252 (16.3)	3556 (17.8)	4.0%
Diabetes with complications	4371 (13.2)	12 440 (19.8)	18.1%	3095 (15.5)	3448 (17.2)	4.8%
Anemia	4225 (12.7)	12 564 (20.0)	19.9%	3136 (15.7)	3797 (19.0)	8.7%
Peripheral vascular disease	4134 (12.5)	11 265 (18.0)	15.4%	2970 (14.9)	3319 (16.6)	4.8%
Cerebrovascular disease	3962 (11.9)	11 350 (18.1)	17.3%	2926 (14.6)	3152 (15.8)	3.1%
Asthma	3727 (11.2)	6701 (10.7)	1.7%	2315 (11.6)	2217 (11.1)	1.5%
Depression	3425 (10.3)	6301 (10.1)	0.9%	2089 (10.5)	2159 (10.8)	1.1%
Anxiety	3381 (10.2)	4250 (6.8)	12.2%	1867 (9.3)	1632 (8.2)	4.2%
Renal disease	3236 (9.8)	12 218 (19.5)	27.8%	2618 (13.1)	3295 (16.5)	9.6%
Myocardial infarction	2736 (8.2)	6423 (10.2)	6.9%	1881 (9.4)	2184 (10.9)	5.0%
Baseline procedures, n (%)						
Gastric bypass surgery	135 (0.4)	215 (0.3)	1.0%	79 (0.4)	86 (0.4)	0.5%
Catheter ablation	1622 (4.9)	2066 (3.3)	8.0%	829 (4.1)	741 (3.7)	2.3%
Coronary bypass graft	477 (1.4)	2405 (3.8)	15.0%	449 (2.2)	653 (3.3)	6.2%
Percutaneous coronary intervention	838 (2.5)	1715 (2.7)	1.3%	599 (3.0)	672 (3.4)	2.1%
Cardioversion	609 (1.8)	870 (1.4)	3.6%	389 (1.9)	349 (1.7)	1.5%
Baseline medication use, n (%)						
Nonoral anticoagulants	3735 (11.3)	7974 (12.7)	4.5%	2275 (11.4)	2436 (12.2)	2.5%
Antihyperlipidemics	2478 (7.5)	7721 (12.3)	16.3%	1843 (9.2)	2053 (10.3)	3.5%
Antihypertensives	30 259 (91.2)	59 387 (94.7)	14.0%	18 442 (92.3)	18 467 (92.4)	0.5%
Antiplatelet agents	3640 (11.0)	6480 (10.3)	2.0%	2459 (12.3)	2406 (12.0)	0.8%
Antiarrhythmia agents	27 435 (82.7)	54 880 (87.6)	13.8%	16 726 (83.7)	16 751 (83.8)	0.3%
Beta‐blockers	22 129 (66.7)	46 080 (73.5)	15.0%	13 682 (68.4)	13 790 (69.0)	1.2%
Calcium channel blockers	12 015 (36.2)	23 884 (38.1)	3.9%	7319 (36.6)	7336 (36.7)	0.2%
Digoxin	2276 (6.9)	12 014 (19.2)	37.2%	1920 (9.6)	2157 (10.8)	3.9%
Baseline all‐cause health care resource use counts, median (IQR)						
Inpatient hospitalization	1 (0–1)	1 (0–2)	16.0%	1 (0–1)	1 (0–1)	2.9%
ED visit	0 (0–0)	0 (0–1)	3.9%	0 (0–0)	0 (0–0)	1.4%
Office visit	8 (5–14)	12 (6–19)	37.4%	9 (5–15)	10 (5–16)	4.8%
Outpatient visit	36 (19–66)	48 (26–84)	25.4%	38 (20–71)	43 (23–75)	6.9%
Pharmacy fill	31 (18–49)	42 (28–63)	40.1%	34 (20–53)	36 (22–55)	3.9%
SNF/long‐term care (yes), n (%)	1279 (3.9)	4002 (6.4)	11.5%	940 (4.7)	1157 (5.8)	4.9%
Baseline costs ($), median (IQR)						
Inpatient hospitalization	3174.28 (0–16 616)	140.54 (0–20 781)	12.0%	3817.61 (0–20 715)	4311.16 (0–25 517)	8.5%
ED visit	0 (0–0)	0 (0–111)	4.1%	0 (0–0)	0 (0–0)	0.1%
Office visit	1006.99 (586–1639)	1206.34 (680–1974)	15.9%	1071.87 (617–1755)	1090.68 (613–1789)	2.5%
Outpatient visit	3518.09 (1229–9417)	3944.15 (1431–10 204)	7.0%	3702.06 (1303–9799)	3801.88 (1339–10 103)	4.3%
Pharmacy fill	2195.61 (631–5618)	3214.07 (1317–6575)	5.2%	2689.6 (886–6089)	2715.54 (889–6135)	2.9%
SNF visit	0 (0–0)	0 (0–0)	9.8%	0 (0–0)	0 (0–0)	2.5%

BMI indicates body mass index; ED, emergency department; IQR, interquartile range; and SNF, skilled nursing facility.

* A standardized mean difference <10% was considered a negligible imbalance.

^†^ 10% or more in either treatment cohort before matching.

Propensity score matching resulted in 19 990 patients with NVAF, obesity, and polypharmacy in each cohort (Table 1). Baseline characteristics of the subgroups by number of concurrent medications, BMI, index year of 2014 and after, and a baseline obesity diagnosis code are provided in Tables S1 through S8. Mean (SD) follow‐up was 2.18 (1.80) years in the rivaroxaban cohort and 2.14 (1.80) years in the warfarin cohort, and median (interquartile range) follow‐up was 1.75 (0.72–3.34) years and 1.72 (0.74–3.27) years, respectively. Patient characteristics were balanced between cohorts after matching, with standardized mean differences <10% for all parameters.

### Outcomes

#### All‐Cause HRU and Costs

**Figure 2:**
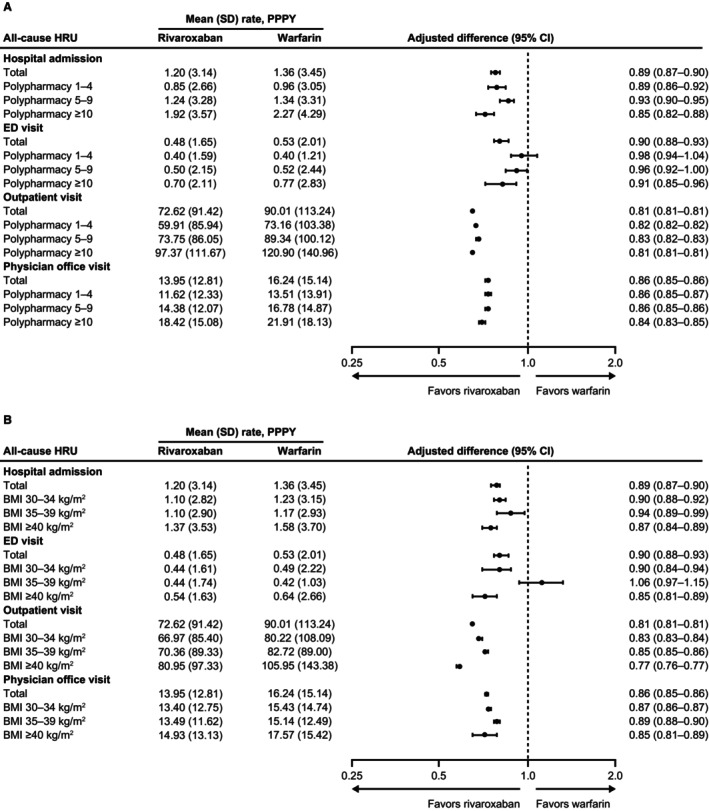
All‐cause HRU for patients with NVAF, polypharmacy, and obesity newly initiating rivaroxaban or warfarin. Data are provided for subgroups by (**A**) number of concurrent medications and (**B**) BMI categories. BMI indicates body mass index; ED, emergency department; HRU, health care resource use; NVAF, nonvalvular atrial fibrillation; and PPPY, per patient per year.

Mean all‐cause HRU rates were lower for the rivaroxaban cohort compared with the warfarin cohort, with rate ratios significantly favoring rivaroxaban for all settings in the overall population (Figure 2). Among patients who had been hospitalized, hospital stay was significantly shorter, by 3 days, with rivaroxaban versus warfarin (mean [SD]: 13.10 [29.37] versus 16.20 [34.46] days; mean difference, −3.10 [95% CI, −3.56 to −2.62]; *P*<0.0001). All‐cause HRU rates for hospital admissions, outpatient visits, and physician office visits were also lower with rivaroxaban versus warfarin for all subgroups by the number of concurrent medications and obesity categories (Figure 2). Length of stay for hospitalized patients was significantly shorter, by ~2 to 3 days, for patients receiving rivaroxaban compared with warfarin in all subgroups except those with a BMI ≥40 kg/m^2^, where the difference in length of stay was −4.36 (−5.20 to −3.46) days. The 30‐day rehospitalization rates were significantly lower with rivaroxaban versus warfarin for all patients and among patients receiving 1 to 4 or ≥10 concurrent medications and those with a BMI of 30 to 34 kg/m^2^ or ≥40 kg/m^2^ (Table 2).

**Table 2:** Rates of 30‐Day Rehospitalization During Follow‐Up

Patients, n (%)	Rivaroxaban	Warfarin	*P* value
Overall (n=19 990 in each arm)	3896 (19.5)	4195 (21.0)	0.0002
Concurrent medications on the index date: 1 to 4 (n=7593 in each arm)	992 (13.1)	1117 (14.7)	0.0034
Concurrent medications on the index date: 5 to 9 (n=8626 in each arm)	1800 (20.9)	1882 (21.8)	0.1276
Concurrent medications on the index date: ≥10 (n=2992 in each arm)	931 (31.1)	1004 (33.6)	0.0436
BMI: 30 to 34 kg/m^2^ (n=9858 in each arm)	1834 (18.6)	1966 (19.9)	0.0172
BMI: 35 to 39 kg/m^2^ (n=2643 in each arm)	487 (18.4)	513 (19.4)	0.3612
BMI: ≥40 kg/m^2^ (n=6849 in each arm)	1426 (20.8)	1599 (23.3)	0.0004
Index year of 2014 and after (n=10 133 in each arm)	1928 (19.0)	2121 (20.9)	0.0007
Obesity based on baseline obesity diagnosis (n=6331 in each arm)	985 (15.6)	1158 (18.3)	0.0007

BMI indicates body mass index.

Sensitivity analyses for all‐cause HRU showed consistent results with significantly lower use in the rivaroxaban cohort versus the warfarin cohort (Table S9). Notably, length of hospitalization was 7 days shorter with rivaroxaban versus warfarin based on the on‐treatment approach.

**Figure 3:**
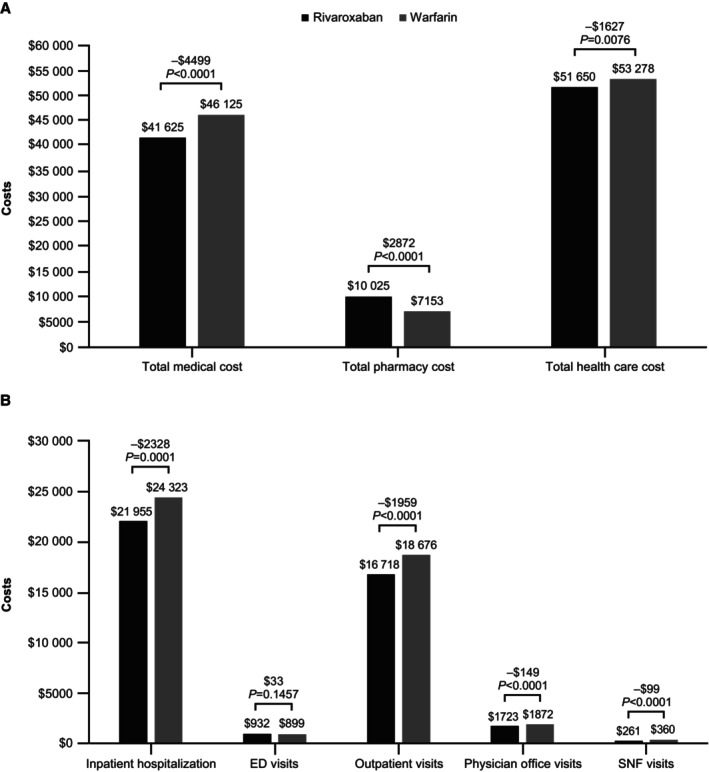
All‐cause (A) total health care costs (medical+pharmacy) and (B) total medical costs for patients with NVAF, polypharmacy, and obesity. Costs are PPPY. ED indicates emergency department; NVAF, nonvalvular atrial fibrillation; PPPY, per patient per year; and SNF, skilled nursing facility.

Mean all‐cause total health care (medical+pharmacy) costs were significantly lower by –$1627 (−$2790 to −$438) PPPY with rivaroxaban versus warfarin (Figure 3A). All‐cause total medical costs difference was –$4499 (−$5660 to −$3305) PPPY and was supported by lower costs in all settings except for ED visits, where the cost difference was not significantly different (Figure 3B). Outpatient pharmacy costs were $2872 ($2670–$3077) PPPY higher for patients treated with rivaroxaban compared with warfarin. When analyzed by subgroups, all‐cause total health care costs were significantly lower for rivaroxaban versus warfarin in patients with 5 to 9 concurrent medications and patients with a BMI ≥40 kg/m^2^ (Table 3). In all other subgroups, there were no significant differences between treatment groups for total health care costs. Total medical costs were more consistently reduced with rivaroxaban versus warfarin in the subgroups except for patients receiving ≥10 concurrent medications, in which the cost difference was not significant. Sensitivity analyses of all‐cause medical costs showed consistent findings with the overall analysis, with results favoring rivaroxaban over warfarin, but total health care costs were higher in the rivaroxaban cohort compared with the warfarin cohort in the on‐treatment analysis (Table S10).

**Table 3:** All‐Cause Health Care Costs (2020 US Dollars) for Subgroups of Patients by Number of Concurrent Medications, Obesity Category, and an Index Year of 2014 and After

Mean (SD) costs, PPPY	Rivaroxaban	Warfarin	Adjusted difference (95% CI)	*P* value
Concurrent medications on the index date: 1–4 (n=7593 in each arm)				
Total medical cost	32 211 (97 641)	34 625 (106 328)	−2414 (−3881 to −877)	0.0024
Inpatient visit cost	15 211 (84 442)	17 678 (85 035)	−2468 (−3735 to −1085)	0.0007
ED cost	869 (5545)	666 (2107)	203 (137 to 275)	<0.0001
Outpatient visit cost	14 484 (42 013)	14 513 (48 641)	−29 (−701 to 676)	0.9346
Physician office visit cost	1483 (2819)	1564 (1926)	−81 (−132 to −28)	0.0029
SNF cost	166 (1944)	207 (1947)	−41 (−54 to −26)	<0.0001
Outpatient pharmacy cost	7107 (12 510)	4406 (12 620)	2701 (2464 to 2946)	<0.0001
Total health care cost	39 319 (99 753)	39 031 (107 690)	287 (−1172 to 1803)	0.7040
Concurrent medications on the index date: 5–9 (n=8626 in each arm)				
Total medical cost	40 875 (107 126)	45 824 (134 525)	−4948 (−6635 to −3189)	<0.0001
Inpatient visit cost	20 924 (85 168)	23 715 (110 221)	−2792 (−4350 to −1107)	0.0015
ED cost	986 (7840)	841 (3916)	144 (75 to 219)	<0.0001
Outpatient visit cost	16 954 (57 500)	19 013 (61 283)	−2060 (−2780 to −1307)	<0.0001
Physician office visit cost	1741 (1774)	1925 (2642)	−184 (−238 to −128)	<0.0001
SNF cost	275 (3091)	331 (2600)	−56 (−77 to −33)	<0.0001
Outpatient pharmacy cost	10 196 (15 279)	7378 (15 265)	2817 (2532 to 3111)	<0.0001
Total health care cost	51 017 (109 067)	53 202 (136 771)	−2131 (−3803 to −403)	0.0160
Concurrent medications on the index date: ≥10 (n=2992 in each arm)				
Total medical cost	68 344 (258 576)	70 916 (145 675)	−2572 (−7164 to 2350)	0.2978
Inpatient visit cost	42 166 (246 483)	40 172 (106 737)	1995 (−2805 to 7411)	0.4319
ED cost	1306 (4354)	1374 (7444)	−68 (−218 to 102)	0.4152
Outpatient visit cost	22 165 (68 042)	26 084 (80 585)	−3919 (−5453 to −2271)	<0.0001
Physician office visit cost	2239 (2524)	2542 (3047)	−303 (−424 to −175)	<0.0001
SNF cost	469 (2935)	754 (7454)	−285 (−345 to −215)	<0.0001
Outpatient pharmacy cost	16 668 (19 506)	13 383 (18 649)	3284 (2568 to 4033)	<0.0001
Total health care cost	85 012 (259 392)	84 300 (147 793)	712 (−3719 to 5387)	0.7581
BMI: 30 to 34 kg/m^2^ (n=9858 in each arm)				
Total medical cost	37 857 (146 721)	39 758 (103 333)	−1901 (−3390 to −351)	0.0167
Inpatient visit cost	19 515 (138 448)	19 820 (75 060)	−305 (−1693 to 1189)	0.6801
ED cost	879 (6913)	803 (3959)	76 (17 to 139)	0.0102
Outpatient visit cost	15 599 (39 411)	17 094 (57 608)	−1495 (−2123 to −840)	<0.0001
Physician office visit cost	1644 (2005)	1747 (1995)	−103 (−153 to −53)	<0.0001
SNF cost	224 (2832)	296 (2316)	−72 (−88 to −54)	<0.0001
Outpatient pharmacy cost	8822 (13 184)	6179 (14 991)	2643 (2394 to 2900)	<0.0001
Total health care cost	46 679 (148 055)	45 937 (105 476)	742 (−742 to 2275)	0.3312
BMI: 35 to 39 kg/m^2^ (n=2643 in each arm)				
Total medical cost	38 102 (89 645)	40 159 (99 180)	−2057 (−4887 to 1000)	0.1819
Inpatient visit cost	19 359 (72 589)	20 617 (70 959)	−1258 (−3821 to 1695)	0.3847
ED cost	823 (6204)	646 (1997)	177 (75 to 292)	0.0003
Outpatient visit cost	16 006 (45 529)	16 703 (55 683)	−697 (−1916 to 623)	0.2916
Physician office visit cost	1646 (1788)	1849 (4733)	−203 (−299 to −101)	0.0001
SNF cost	270 (2217)	346 (2308)	−76 (−113 to −34)	0.0008
Outpatient pharmacy cost	10 712 (16 852)	7291 (15 394)	3421 (2851 to 4024)	<0.0001
Total health care cost	48 814 (93 709)	47 450 (101 026)	1365 (−1496 to 4404)	0.3575
BMI: ≥40 kg/m^2^ (n=6849 in each arm)				
Total medical cost	48 557 (132 388)	57 629 (216 597)	−9072 (−11 361 to −6669)	<0.0001
Inpatient visit cost	27 388 (119 188)	33 605 (203 246)	−6217 (−8507 to −3717)	<0.0001
ED cost	1028 (3666)	1110 (5751)	−81 (−163 to 7)	0.0705
Outpatient visit cost	18 012 (46 263)	20 426 (57 986)	−2414 (−3263 to −1523)	<0.0001
Physician office visit cost	1844 (2142)	2037 (2265)	−192 (−258 to −125)	<0.0001
SNF cost	287 (2457)	458 (5096)	−170 (−195 to −143)	<0.0001
Outpatient pharmacy cost	11 129 (16 136)	8461 (16 371)	2668 (2297 to 3053)	<0.0001
Total health care cost	59 686 (134 102)	66 090 (217 951)	−6404 (−8681 to −4036)	<0.0001

BMI indicates body mass index; ED, emergency department; PPPY, per patient per year; and SNF, skilled nursing facility.

#### NVAF‐Related HRU and Costs

NVAF‐related HRU rates followed a similar pattern as all‐cause HRU rates, with rate ratios for all settings significantly favoring rivaroxaban compared with warfarin (Table S11). Mean difference in the length of hospital stay for NVAF‐related hospital admissions was shorter with rivaroxaban versus warfarin (−3.12 [−3.56 to −2.65] days; *P*<0.0001). NVAF‐related HRU rates were lower with rivaroxaban versus warfarin for all subgroups by the number of concurrent medications and obesity categories, with significant differences consistently achieved for length of hospital stay, outpatient visits, and physician office visits, but not for ED visits (Table S11). Hospital admissions were not significantly different for the subgroup of patients receiving 1 to 4 concurrent medications or patients with a BMI of 35 to 39 kg/m^2^. Length of hospital stay was significantly shorter for patients receiving rivaroxaban versus warfarin, by approximately 1 to 3 days for NVAF‐related hospitalizations in all subgroups analyzed and by nearly 5 days for patients with BMI of ≥40 kg/m^2^.

Mean total medical costs related to NVAF were not significantly different between the rivaroxaban and warfarin cohorts, with an adjusted difference of $144 (−$756 to $1079) PPPY (Table S12). Compared with patients receiving warfarin, patients receiving rivaroxaban had significantly higher NVAF‐related costs associated with outpatient visits and significantly lower costs associated with inpatient visits, physician office visits, and skilled nursing facility costs.

NVAF‐related total medical costs in concurrent medication and obesity subgroups were generally similar for rivaroxaban compared with warfarin, with significant differences observed for patients in the BMI subgroups.

Sensitivity analyses for NVAF‐related HRU showed generally consistent results with significantly lower HRU rates with rivaroxaban versus warfarin (Table S12). NVAF‐related total medical costs were not significantly different between the 2 anticoagulants based on the analysis by index year of 2014 and after or when obesity was based on a baseline diagnosis. Using the on‐treatment approach, NVAF‐related medical costs were significantly higher with rivaroxaban versus warfarin ($2023 [$1465–$2604] PPPY).

## DISCUSSION

In this real‐world analysis of patients with NVAF, polypharmacy, and obesity, rivaroxaban was associated with significantly lower rates of HRU compared with warfarin. The effect was generally consistent for all‐cause and NVAF‐related HRU.

The comparative effectiveness and safety of rivaroxaban versus warfarin have been demonstrated in clinical and real‐world studies of patients with polypharmacy and obesity and may provide insight into the economic outcomes assessed in this study. In an analysis of the ROCKET‐AF study, 36% of patients with NVAF were receiving 0 to 4 concurrent medications, 51% were receiving 5 to 9 medications, and 13% were receiving ≥10 medications.^19^ Although polypharmacy was associated with a higher risk of the composite endpoint of stroke, non–central nervous system systemic embolism, vascular death, or myocardial infarction, the efficacy and safety of rivaroxaban were not different between subgroups of patients by number of concurrent medications; however, patients receiving ≥5 concurrent medications had a higher risk of bleeding than those on 0 to 4 concurrent medications.

Among patients with morbid obesity and NVAF (ie, BMI >40 kg/m^2^) in 2 health care claims databases, the risks of ischemic stroke or systemic embolism and major bleeding were similar between rivaroxaban and warfarin and the use of rivaroxaban was associated with significantly reduced total health care costs.^29^ In a retrospective chart review including 176 patients with NVAF who had a BMI >40 kg/m^2^ or a body weight >120 kg, venous thromboembolism recurrence, stroke, or death occurred within 12 months of treatment initiation in 5% of patients receiving rivaroxaban and 13% of patients receiving warfarin.^30^ The length of stay was significantly shorter for the rivaroxaban group versus the warfarin group (2 versus 4 days), and bleeding complications were not significantly different. In another study using the same health care claims databases, among patients with NVAF, obesity, and polypharmacy, rivaroxaban was associated with a lower risk of stroke and systemic embolism and major bleeding risk was not significantly different between rivaroxaban and warfarin overall or across subgroups by number of concurrent medications.^31^

In the current analysis, overall all‐cause total health care costs and total medical costs were significantly lower for patients receiving rivaroxaban compared with warfarin, with reductions of $1627 and $4499 PPPY, respectively. These results are consistent with lower all‐cause HRU rates with rivaroxaban versus warfarin and may be influenced by the induction time of warfarin during hospitalization and the monitoring required with the use of warfarin. However, despite the lower NVAF‐related HRU rates associated with rivaroxaban use, NVAF‐related total medical costs were not significantly different, which may be related to the higher outpatient visit costs with rivaroxaban. The observed differences between lower NVAF‐related HRU rates and higher NVAF‐related costs may be due to unobserved confounding in this study because the NVAF‐related cost findings are not consistent with other real‐world evidence studies conducted in patients with obesity and NVAF.^29^, ^30^, ^31^, ^32^, ^33^, ^34^, ^35^

Rehospitalization rates were significantly lower with rivaroxaban versus warfarin overall and in key subgroups, with the following 2 exceptions: patients who were taking 5 to 9 concurrent medications on the index date and patients with a BMI of 35 to 39 kg/m^2^. In these subgroups, there was no statistical difference in the proportion of patients who were rehospitalized within 30 days, but the risk was numerically smaller with rivaroxaban versus warfarin. The explanation for this finding is unclear, especially since the specific concurrent medications being used were not discerned in the analysis. A possible explanation is less control over comorbidities in those receiving 5 to 9 concurrent medications, leading to rehospitalization. Additionally, there is no clear reason for the trend correlating with BMI, but the BMI of 35 to 39 kg/m^2^ category had the fewest patients and a smaller number of patients needing rehospitalization, which may have made it difficult to observe a statistical difference.

In a recent real‐world study using IQVIA PharMetrics Plus data for patients with NVAF and obesity, those receiving rivaroxaban had lower all‐cause and NVAF‐related HRU and medical costs compared with those receiving warfarin.^32^ NVAF‐related total medical costs were significantly reduced with rivaroxaban versus warfarin in the up to 12‐month (−$3352) and up to 36‐month (−$1829) analyses among patients with NVAF and obesity. The medical cost savings were driven by lower hospitalization costs, which offset the higher pharmacy costs associated with rivaroxaban. Using the same database, Laliberté and colleagues identified patients with NVAF, obesity, and polypharmacy.^33^ The rivaroxaban cohort had a significantly lower mean rate of HRU compared with the warfarin cohort across all settings at 12 months and for all‐cause hospitalizations, ED visits, and outpatient visits over >9 months of follow‐up. Rates of NVAF‐related HRU were comparable, with significantly lower hospitalizations, ED visits, and outpatient visits for those receiving rivaroxaban versus those receiving warfarin. Rivaroxaban was also associated with lower all‐cause medical costs, which were driven by lower hospitalization costs, compared with warfarin at both 12 and 36 months of follow‐up. Total health care costs were significantly different between cohorts at 12 months but not at 36 months. NVAF‐related medical costs were numerically, but not significantly, lower for patients receiving rivaroxaban in the up to 12‐month (*−*$2621) and up to 36‐month (−$1152) analyses for patients with obesity and NVAF and who were receiving ≥5 concurrent medications.

Diabetes was a common comorbidity in the current study population. The results of a real‐world analysis of HRU and costs among patients with NVAF, obesity, and diabetes using the Optum database provide additional perspective. In approximately 20 000 patients with NVAF receiving rivaroxaban or warfarin who had concurrent obesity and diabetes, the rivaroxaban cohort had reduced all‐cause HRU in all settings except ED visits.^34^ NVAF‐related HRU was also significantly lower with rivaroxaban versus warfarin, especially for physician office visits and hospital outpatient visits. Rivaroxaban was also associated with a reduction of approximately $1100 PPPY in NVAF‐related total medical costs versus warfarin.

There are several potential explanations for the differences in NVAF‐related costs across studies. The current analysis used the MarketScan combined database, which included 52% of commercial claims and 48% of supplemental Medicare claims. In contrast, the prior studies used the 100% commercial IQVIA PharMetrics Plus database and 75% Medicare Optum database. The MarketScan Medicare database consists of claims for supplemental coverage and may represent a patient population that is generally healthier and less likely to be hospitalized. It is also possible that some costs in the MarketScan database were not attributed to NVAF and thus not captured in this analysis.

The use of both commercially insured and Medicare supplemental databases across a geographically diverse area may have captured beneficiaries across the United States but not those using Medicare without supplemental insurance. Our study has several other limitations are noting. The use of administrative claims data is subject to potential coding errors and inconsistencies. Propensity score matching reduces confounding but only on observed variables; thus, there may be residual confounding for characteristics that were not identified (such as disease severity that is not well captured by the diagnosis codes). Other factors that may contribute to general misclassification include the fact that patients' BMIs were not provided directly in claims data, and we used machine‐learning–based algorithms to calculate BMI and create 3 BMI categories. Although international normalized ratio monitoring and costs are captured, the time in the therapeutic international normalized ratio for patients treated with warfarin was not assessed, leading to potential under‐ or overtreatment with warfarin in patients that could affect outcomes. Moreover, claims for prescription medication do not assure that the medication was taken as prescribed, and over‐the‐counter medications, such as aspirin, or medication samples provided by physicians were not captured in the claims data. The use of the 14‐day grace period for defining polypharmacy was intended to increase positive predictive value but was an arbitrary selection; some medications with longer grace periods could have been missed. Cost analyses do not capture out‐of‐pocket expenses or indirect costs, such as potential lost wages from disability. Despite our methods to reduce the risk of data misclassification, these limitations could lead to overestimation of benefits in this observational analysis.

## CONCLUSIONS

For a high‐risk cohort of patients with NVAF, polypharmacy, and obesity, rivaroxaban was associated with lower all‐cause and NVAF‐related HRU and lower all‐cause medical and total health care costs compared with warfarin.

## Sources of Funding

This study was funded by Janssen Scientific Affairs, LLC (Titusville, NJ, USA).

## Disclosures

Mark Alberts has not received compensation for this project but received consultancy fees from Janssen Pharmaceuticals and Janssen Scientific Affairs for being on the steering committee of the Quantum AF study. Jinghua He, Akshay Kharat, Christopher D. Pericone, and Veronica Ashton are full‐time employees of Janssen Scientific Services, LLC, a Johnson & Johnson Company. Merative and MarketScan are trademarks of Merative Corporation in the United States, other countries, or both.

## Supporting information

Data S1–S3
Tables S1–S12
Figures S1–S2
