# Supplementary material for: Health Care Resource Use and Costs of Rivaroxaban Versus Warfarin Among Nonvalvular Atrial Fibrillation Polypharmacy Patients With Obesity
Source: J Am Heart Assoc. 2025 Jan 10;14(2):e036401. doi: 10.1161/JAHA.124.036401 (PMC12054406; doi:10.1161/JAHA.124.036401)

Supplemental Material

Data S1. Diagnosis and procedure codes for exclusion criteria

Diagnosis Codes for Mitral-stenosis and Related Disorders	
**Diagnosis Code**	**Definition**
**ICD-9-CM**	
394.0x	Mitral stenosis
394.2x	Mitral stenosis with insufficiency
396.0x	Mitral valve stenosis and aortic valve stenosis
396.1x	Mitral valve stenosis and aortic valve insufficiency
396.8x	Multiple involvement of mitral and aortic valves
396.9x	Mitral and aortic valve diseases, unspecified
746.5x	Congenital mitral stenosis
996.02	Mechanical complication due to heart valve prosthesis
996.71	Other complications; due to heart valve prosthesis
**ICD-10-CM**	
I05.0x	Rheumatic mitral stenosis
I05.2x	Rheumatic mitral stenosis with insufficiency
I08.0x	Rheumatic disorders of both mitral and aortic valves
I08.1x	Rheumatic disorders of both mitral and tricuspid valves
I08.3x	Combined rheumatic disorders of mitral, aortic and tricuspid valves
I08.8x	Other rheumatic multiple valve diseases
I08.9x	Rheumatic multiple valve disease, unspecified
I34.2x	Nonrheumatic mitral (valve) stenosis
Q23.2x	Congenital mitral stenosis
T82.0x	Mechanical complication of heart valve prosthesis
T82.22x	Mechanical complication of biological heart valve graft
T82.827x	Fibrosis due to cardiac prosthetic devices, implants and grafts
T82.837x	Hemorrhage due to cardiac prosthetic devices, implants and grafts
T82.847x	Pain due to cardiac prosthetic devices, implants and grafts
T82.857x	Stenosis of other cardiac prosthetic devices, implants and grafts
T82.867x	Thrombosis due to cardiac prosthetic devices, implants and grafts
T82.897x	Other specified complication of cardiac prosthetic devices, implants and grafts
T82.9xx	Unspecified complication of cardiac and vascular prosthetic device, implant and graft

ICD-9-CM: *International Classification of Diseases, 9th Revision, Clinical Modification*; ICD-10-CM: *International Classification of Diseases, 10th Revision, Clinical Modification*.

ICD-9-CM Diagnosis Codes for VTE Diagnosis	
**Diagnosis Code**	**Definition**
**PE**	
415.1	Pulmonary embolism and infarction
415.11	Iatrogenic pulmonary embolism and infarction
415.12	Septic pulmonary embolism
415.13	Saddle embolus of pulmonary artery
415.19	Other pulmonary embolism and infarction
**DVT**	
451.1	Phlebitis and thrombophlebitis of deep veins of lower extremities
451.11	Phlebitis and thrombophlebitis of femoral vein (deep) (superficial)
451.19	Phlebitis and thrombophlebitis of other deep vessels of lower extremities
451.2	Phlebitis and thrombophlebitis of lower extremities, unspecified
453.4	Acute venous embolism and thrombosis of deep vessels of lower extremity
453.40	Acute venous embolism and thrombosis of unspecified deep vessels of lower extremity
453.41	Acute venous embolism and thrombosis of deep vessels of proximal lower extremity
453.42	Acute venous embolism and thrombosis of deep vessels of distal lower extremity
453.8	Acute venous embolism and thrombosis of other specified veins
453.81	Acute venous embolism and thrombosis of superficial veins of upper extremity
453.82	Acute venous embolism and thrombosis of deep veins of upper extremity
453.83	Acute venous embolism and thrombosis of upper extremity, unspecified
453.84	Acute venous embolism and thrombosis of axillary veins
453.85	Acute venous embolism and thrombosis of subclavian veins
453.86	Acute venous embolism and thrombosis of internal jugular veins
453.87	Acute venous embolism and thrombosis of other thoracic veins
453.89	Acute venous embolism and thrombosis of other specified veins
453.9	Embolism and thrombosis of unspecified site

ICD-9-CM: *International Classification of Diseases, 9th Revision, Clinical Modification*; VTE: venous thromboembolism; PE: pulmonary embolism; DVT: deep vein thrombosis; ICD: *International Classification of Diseases*.

ICD-10-CM Diagnosis Codes for VTE Diagnosis	
**Diagnosis Code**	**Full Description**
**PE**	
**I26**	**Pulmonary embolism**
I26.0	Pulmonary embolism with acute cor pulmonale
I26.01	Septic pulmonary embolism with acute cor pulmonale
I26.02	Saddle embolus of pulmonary artery with acute cor pulmonale
I26.09	Other pulmonary embolism with acute cor pulmonale
I26.9	Pulmonary embolism without acute cor pulmonale
I26.90	Septic pulmonary embolism without acute cor pulmonale
I26.92	Saddle embolus of pulmonary artery without acute cor pulmonale

I26.99		Other pulmonary embolism without acute cor pulmonale
**DVT**		
**I80**		**Phlebitis and thrombophlebitis**
I80.1		Phlebitis and thrombophlebitis of femoral vein
I80.10		Phlebitis and thrombophlebitis of unspecified femoral vein
I80.11		Phlebitis and thrombophlebitis of right femoral vein
I80.12		Phlebitis and thrombophlebitis of left femoral vein
I80.13		Phlebitis and thrombophlebitis of femoral vein, bilateral
I80.2		Phlebitis and thrombophlebitis of other and unspecified deep vessels of lower extremities
I80.20		Phlebitis and thrombophlebitis of unspecified deep vessels of lower extremities
	I80.201	Phlebitis and thrombophlebitis of unspecified deep vessels of right lower extremity
	I80.202	Phlebitis and thrombophlebitis of unspecified deep vessels of left lower extremity
	I80.203	Phlebitis and thrombophlebitis of unspecified deep vessels of lower extremities, bilateral
	I80.209	Phlebitis and thrombophlebitis of unspecified deep vessels of unspecified lower extremity
I80.21		Phlebitis and thrombophlebitis of iliac vein
	I80.211	Phlebitis and thrombophlebitis of right iliac vein
	I80.212	Phlebitis and thrombophlebitis of left iliac vein
	I80.213	Phlebitis and thrombophlebitis of iliac vein, bilateral
	I80.219	Phlebitis and thrombophlebitis of unspecified iliac vein
I80.22		Phlebitis and thrombophlebitis of popliteal vein
	I80.221	Phlebitis and thrombophlebitis of right popliteal vein
	I80.222	Phlebitis and thrombophlebitis of left popliteal vein
	I80.223	Phlebitis and thrombophlebitis of popliteal vein, bilateral
	I80.229	Phlebitis and thrombophlebitis of unspecified popliteal vein
I80.23		Phlebitis and thrombophlebitis of tibial vein
	I80.231	Phlebitis and thrombophlebitis of right tibial vein
	I80.232	Phlebitis and thrombophlebitis of left tibial vein
	I80.233	Phlebitis and thrombophlebitis of tibial vein, bilateral
	I80.239	Phlebitis and thrombophlebitis of unspecified tibial vein
I80.29		Phlebitis and thrombophlebitis of other deep vessels of lower extremities
	I80.291	Phlebitis and thrombophlebitis of other deep vessels of right lower extremity
	I80.292	Phlebitis and thrombophlebitis of other deep vessels of left lower extremity
	I80.293	Phlebitis and thrombophlebitis of other deep vessels of lower extremity, bilateral
	I80.299	Phlebitis and thrombophlebitis of other deep vessels of unspecified lower extremity
I80.3		Phlebitis and thrombophlebitis of lower extremities, unspecified
**I82**		**Other venous embolism and thrombosis**
I82.4		Acute embolism and thrombosis of deep veins of lower extremity
I82.40		Acute embolism and thrombosis of unspecified deep veins of lower extremity
	I82.401	Acute embolism and thrombosis of unspecified deep veins of right lower extremity
	I82.402	Acute embolism and thrombosis of unspecified deep veins of left lower extremity
	I82.403	Acute embolism and thrombosis of unspecified deep veins of lower extremity, bilateral
	I82.409	Acute embolism and thrombosis of unspecified deep veins of unspecified lower extremity
I82.41		Acute embolism and thrombosis of femoral vein
	I82.411	Acute embolism and thrombosis of right femoral vein
	I82.412	Acute embolism and thrombosis of left femoral vein
	I82.413	Acute embolism and thrombosis of femoral vein, bilateral
	I82.419	Acute embolism and thrombosis of unspecified femoral vein
I82.42		Acute embolism and thrombosis of iliac vein
	I82.421	Acute embolism and thrombosis of right iliac vein

I82.422	Acute embolism and thrombosis of left iliac vein
I82.423	Acute embolism and thrombosis of iliac vein, bilateral
I82.429	Acute embolism and thrombosis of unspecified iliac vein
I82.43	Acute embolism and thrombosis of popliteal vein
I82.431	Acute embolism and thrombosis of right popliteal vein
I82.432	Acute embolism and thrombosis of left popliteal vein
I82.433	Acute embolism and thrombosis of popliteal vein, bilateral
I82.439	Acute embolism and thrombosis of unspecified popliteal vein
I82.44	Acute embolism and thrombosis of tibial vein
I82.441	Acute embolism and thrombosis of right tibial vein
I82.442	Acute embolism and thrombosis of left tibial vein
I82.443	Acute embolism and thrombosis of tibial vein, bilateral
I82.449	Acute embolism and thrombosis of unspecified tibial vein
I82.49	Acute embolism and thrombosis of other specified deep vein of lower extremity
I82.491	Acute embolism and thrombosis of other specified deep vein of right lower extremity
I82.492	Acute embolism and thrombosis of other specified deep vein of left lower extremity
I82.493	Acute embolism and thrombosis of other specified deep vein of lower extremity, bilateral
I82.499	Acute embolism and thrombosis of other specified deep vein of unspecified lower extremity
I82.4Y	Acute embolism and thrombosis of unspecified deep veins of proximal lower extremity
I82.4Y1	Acute embolism and thrombosis of unspecified deep veins of right proximal lower extremity
I82.4Y2	Acute embolism and thrombosis of unspecified deep veins of left proximal lower extremity
I82.4Y3	Acute embolism and thrombosis of unspecified deep veins of proximal lower extremity, bilateral
I82.4Y9	Acute embolism and thrombosis of unspecified deep veins of unspecified proximal lower extremity
I82.4Z	Acute embolism and thrombosis of unspecified deep veins of distal lower extremity
I82.4Z1	Acute embolism and thrombosis of unspecified deep veins of right distal lower extremity
I82.4Z2	Acute embolism and thrombosis of unspecified deep veins of left distal lower extremity
I82.4Z3	Acute embolism and thrombosis of unspecified deep veins of distal lower extremity, bilateral
I82.4Z9	Acute embolism and thrombosis of unspecified deep veins of unspecified distal lower extremity
I82.6	Acute embolism and thrombosis of veins of upper extremity
I82.60	Acute embolism and thrombosis of unspecified veins of upper extremity
I82.601	Acute embolism and thrombosis of unspecified veins of right upper extremity
I82.602	Acute embolism and thrombosis of unspecified veins of left upper extremity
I82.603	Acute embolism and thrombosis of unspecified veins of upper extremity, bilateral
I82.609	Acute embolism and thrombosis of unspecified veins of unspecified upper extremity
I82.61	Acute embolism and thrombosis of superficial veins of upper extremity
I82.611	Acute embolism and thrombosis of superficial veins of right upper extremity
I82.612	Acute embolism and thrombosis of superficial veins of left upper extremity
I82.613	Acute embolism and thrombosis of superficial veins of upper extremity, bilateral
I82.619	Acute embolism and thrombosis of superficial veins of unspecified upper extremity
I82.62	Acute embolism and thrombosis of deep veins of upper extremity
I82.621	Acute embolism and thrombosis of deep veins of right upper extremity
I82.622	Acute embolism and thrombosis of deep veins of left upper extremity
I82.623	Acute embolism and thrombosis of deep veins of upper extremity, bilateral

I82.629	Acute embolism and thrombosis of deep veins of unspecified upper extremity
I82.A	Embolism and thrombosis of axillary vein
I82.A1	Acute embolism and thrombosis of axillary vein
I82.A11	Acute embolism and thrombosis of right axillary vein
I82.A12	Acute embolism and thrombosis of left axillary vein
I82.A13	Acute embolism and thrombosis of axillary vein, bilateral
I82.A19	Acute embolism and thrombosis of unspecified axillary vein
I82.B	Embolism and thrombosis of subclavian vein
I82.B1	Acute embolism and thrombosis of subclavian vein
I82.B11	Acute embolism and thrombosis of right subclavian vein
I82.B12	Acute embolism and thrombosis of left subclavian vein
I82.B13	Acute embolism and thrombosis of subclavian vein, bilateral
I82.B19	Acute embolism and thrombosis of unspecified subclavian vein
I82.C	Embolism and thrombosis of internal jugular vein
I82.C1	Acute embolism and thrombosis of internal jugular vein
I82.C11	Acute embolism and thrombosis of right internal jugular vein
I82.C12	Acute embolism and thrombosis of left internal jugular vein
I82.C13	Acute embolism and thrombosis of internal jugular vein, bilateral
I82.C19	Acute embolism and thrombosis of unspecified internal jugular vein
I82.90	Acute embolism and thrombosis of unspecified vein

ICD-10-CM: International Classification of Diseases, 10th Revision, Clinical Modification; VTE: venous thromboembolism; PE: pulmonary embolism; DVT: deep vein thrombosis; ICD: International Classification of Diseases.

**Procedure Codes for Hip/Knee Replacement**		
**Procedure Code**	**Definition**	**Code Type**
01214	Anesthesia for open procedures involving hip joint; total hip arthroplasty	CPT
01215	Anesthesia for open procedures involving hip joint; revision of total hip arthroplasty	CPT
27130	Arthroplasty, acetabular and proximal femoral prosthetic replacement (total hip arthroplasty), with or without autograft or allograft	CPT
27132	Conversion of previous hip surgery to total hip arthroplasty, with or without autograft or allograft	CPT
27134	Revision of total hip arthroplasty; both components, with or without autograft or allograft	CPT
00.70	Revision of hip replacement, both acetabular and femoral components	ICD-9-PCS
81.51	Total hip replacement	ICD-9-PCS
0SP908Z	Removal of Spacer from Right Hip Joint, Open Approach	ICD-10-PCS
0SP909Z	Removal of Liner from Right Hip Joint, Open Approach	ICD-10-PCS
0SP90BZ	Removal of Resurfacing Device from Right Hip Joint, Open Approach	ICD-10-PCS
0SP90JZ	Removal of Synthetic Substitute from Right Hip Joint, Open Approach	ICD-10-PCS

0SPB08Z	Removal of Spacer from Left Hip Joint, Open Approach	ICD-10-PCS
0SPB09Z	Removal of Liner from Left Hip Joint, Open Approach	ICD-10-PCS
0SPB0BZ	Removal of Resurfacing Device from Left Hip Joint, Open Approach	ICD-10-PCS
0SPB0JZ	Removal of Synthetic Substitute from Left Hip Joint, Open Approach	ICD-10-PCS
0SR9019	Replacement of Right Hip Joint with Metal Synthetic Substitute, Cemented, Open Approach	ICD-10-PCS
0SR901A	Replacement of Right Hip Joint with Metal Synthetic Substitute, Uncemented, Open Approach	ICD-10-PCS
0SR901Z	Replacement of Right Hip Joint with Metal Synthetic Substitute, Open Approach	ICD-10-PCS
0SR9029	Replacement of Right Hip Joint with Metal on Polyethylene Synthetic Substitute, Cemented, Open Approach	ICD-10-PCS
0SR902A	Replacement of Right Hip Joint with Metal on Polyethylene Synthetic Substitute, Uncemented, Open Approach	ICD-10-PCS
0SR902Z	Replacement of Right Hip Joint with Metal on Polyethylene Synthetic Substitute, Open Approach	ICD-10-PCS
0SR9039	Replacement of Right Hip Joint with Ceramic Synthetic Substitute, Cemented, Open Approach	ICD-10-PCS
0SR903A	Replacement of Right Hip Joint with Ceramic Synthetic Substitute, Uncemented, Open Approach	ICD-10-PCS
0SR903Z	Replacement of Right Hip Joint with Ceramic Synthetic Substitute, Open Approach	ICD-10-PCS
0SR9049	Replacement of Right Hip Joint with Ceramic on Polyethylene Synthetic Substitute, Cemented, Open Approach	ICD-10-PCS
0SR904A	Replacement of Right Hip Joint with Ceramic on Polyethylene Synthetic Substitute, Uncemented, Open Approach	ICD-10-PCS
0SR904Z	Replacement of Right Hip Joint with Ceramic on Polyethylene Synthetic Substitute, Open Approach	ICD-10-PCS
0SR9069	Replacement of Right Hip Joint with Oxidized Zirconium on Polyethylene Synthetic Substitute, Cemented, Open Approach	ICD-10-PCS
0SR906A	Replacement of Right Hip Joint with Oxidized Zirconium on Polyethylene Synthetic Substitute, Uncemented, Open Approach	ICD-10-PCS
0SR906Z	Replacement of Right Hip Joint with Oxidized Zirconium on Polyethylene Synthetic Substitute, Open Approach	ICD-10-PCS
0SR90J9	Replacement of Right Hip Joint with Synthetic Substitute, Cemented, Open Approach	ICD-10-PCS
0SR90JA	Replacement of Right Hip Joint with Synthetic Substitute, Uncemented, Open Approach	ICD-10-PCS
0SR90JZ	Replacement of Right Hip Joint with Synthetic Substitute, Open Approach	ICD-10-PCS
0SRB019	Replacement of Left Hip Joint with Metal Synthetic Substitute, Cemented, Open Approach	ICD-10-PCS
0SRB01A	Replacement of Left Hip Joint with Metal Synthetic Substitute, Uncemented, Open Approach	ICD-10-PCS
0SRB01Z	Replacement of Left Hip Joint with Metal Synthetic Substitute, Open Approach	ICD-10-PCS
0SRB029	Replacement of Left Hip Joint with Metal on Polyethylene Synthetic Substitute, Cemented, Open	ICD-10-PCS

0SRB02A	Replacement of Left Hip Joint with Metal on Polyethylene Synthetic Substitute, Uncemented, Open Approach	ICD-10-PCS
0SRB02Z	Replacement of Left Hip Joint with Metal on Polyethylene Synthetic Substitute, Open Approach	ICD-10-PCS
0SRB039	Replacement of Left Hip Joint with Ceramic Synthetic Substitute, Cemented, Open Approach	ICD-10-PCS
0SRB03A	Replacement of Left Hip Joint with Ceramic Synthetic Substitute, Uncemented, Open Approach	ICD-10-PCS
0SRB03Z	Replacement of Left Hip Joint with Ceramic Synthetic Substitute, Open Approach	ICD-10-PCS
0SRB049	Replacement of Left Hip Joint with Ceramic on Polyethylene Synthetic Substitute, Cemented, Open Approach	ICD-10-PCS
0SRB04A	Replacement of Left Hip Joint with Ceramic on Polyethylene Synthetic Substitute, Uncemented, Open Approach	ICD-10-PCS
0SRB04Z	Replacement of Left Hip Joint with Ceramic on Polyethylene Synthetic Substitute, Open Approach	ICD-10-PCS
0SRB069	Replacement of Left Hip Joint with Oxidized Zirconium on Polyethylene Synthetic Substitute, Cemented, Open Approach	ICD-10-PCS
0SRB06A	Replacement of Left Hip Joint with Oxidized Zirconium on Polyethylene Synthetic Substitute, Uncemented, Open Approach	ICD-10-PCS
0SRB06Z	Replacement of Left Hip Joint with Oxidized Zirconium on Polyethylene Synthetic Substitute, Open Approach	ICD-10-PCS
0SRB0J9	Replacement of Left Hip Joint with Synthetic Substitute, Cemented, Open Approach	ICD-10-PCS
0SRB0JA	Replacement of Left Hip Joint with Synthetic Substitute, Uncemented, Open Approach	ICD-10-PCS
0SRB0JZ	Replacement of Left Hip Joint with Synthetic Substitute, Open Approach	ICD-10-PCS
Total Knee Replacement		
01402	Anesthesia for open or surgical arthroscopic procedures on knee joint; total knee arthroplasty	CPT
27447	Arthroplasty, knee, condyle and plateau; medial AND lateral compartments with or without patella resurfacing (total knee arthroplasty)	CPT
27486	Revision of total knee arthroplasty, with or without allograft; 1 component	CPT
27487	Revision of total knee arthroplasty, with or without allograft; femoral and entire tibial component	CPT
00.80	Revision of knee replacement, total (all components)	ICD-9-PCS
00.84	Revision of total knee replacement, tibial insert (liner)	ICD-9-PCS
81.54	Total knee replacement	ICD-9-PCS
81.55	Revision of knee replacement, not otherwise specified	ICD-9-PCS
0SPC08Z	Removal of Spacer from Right Knee Joint, Open Approach	ICD-10-PCS
0SPC09Z	Removal of Liner from Right Knee Joint, Open Approach	ICD-10-PCS
0SPC0JZ	Removal of Synthetic Substitute from Right Knee Joint, Open Approach	ICD-10-PCS
0SPC48Z	Removal of Spacer from Right Knee Joint, Percutaneous Endoscopic Approach	ICD-10-PCS

0SPC4JZ	Removal of Synthetic Substitute from Right Knee Joint, Percutaneous Endoscopic Approach	ICD-10-PCS
0SPD08Z	Removal of Spacer from Left Knee Joint, Open Approach	ICD-10-PCS
0SPD09Z	Removal of Liner from Left Knee Joint, Open Approach	ICD-10-PCS
0SPD0JZ	Removal of Synthetic Substitute from Left Knee Joint, Open Approach	ICD-10-PCS
0SPD48Z	Removal of Spacer from Left Knee Joint, Percutaneous Endoscopic Approach	ICD-10-PCS
0SPD4JZ	Removal of Synthetic Substitute from Left Knee Joint, Percutaneous Endoscopic Approach	ICD-10-PCS
0SRC069	Replacement of Right Knee Joint with Oxidized Zirconium on Polyethylene Synthetic Substitute, Cemented, Open Approach	ICD-10-PCS
0SRC06A	Replacement of Right Knee Joint with Oxidized Zirconium on Polyethylene Synthetic Substitute, Uncemented, Open Approach	ICD-10-PCS
0SRC06Z	Replacement of Right Knee Joint with Oxidized Zirconium on Polyethylene Synthetic Substitute, Open Approach	ICD-10-PCS
0SRC07Z	Replacement of Right Knee Joint with Autologous Tissue Substitute, Open Approach	ICD-10-PCS
0SRC0J9	Replacement of Right Knee Joint with Synthetic Substitute, Cemented, Open Approach	ICD-10-PCS
0SRC0JA	Replacement of Right Knee Joint with Synthetic Substitute, Uncemented, Open Approach	ICD-10-PCS
0SRC0JZ	Replacement of Right Knee Joint with Synthetic Substitute, Open Approach	ICD-10-PCS
0SRC0KZ	Replacement of Right Knee Joint with Nonautologous Tissue Substitute, Open Approach	ICD-10-PCS
0SRC0LZ	Replacement of Right Knee Joint with Unicondylar Synthetic Substitute, Open Approach	ICD-10-PCS
0SRD069	Replacement of Left Knee Joint with Oxidized Zirconium on Polyethylene Synthetic Substitute, Cemented, Open Approach	ICD-10-PCS
0SRD06A	Replacement of Left Knee Joint with Oxidized Zirconium on Polyethylene Synthetic Substitute, Uncemented, Open Approach	ICD-10-PCS
0SRD06Z	Replacement of Left Knee Joint with Oxidized Zirconium on Polyethylene Synthetic Substitute, Open Approach	ICD-10-PCS
0SRD07Z	Replacement of Left Knee Joint with Autologous Tissue Substitute, Open Approach	ICD-10-PCS
0SRD0J9	Replacement of Left Knee Joint with Synthetic Substitute, Cemented, Open Approach	ICD-10-PCS
0SRD0JA	Replacement of Left Knee Joint with Synthetic Substitute, Uncemented, Open Approach	ICD-10-PCS
0SRD0JZ	Replacement of Left Knee Joint with Synthetic Substitute, Open Approach	ICD-10-PCS
0SRD0KZ	Replacement of Left Knee Joint with Nonautologous Tissue Substitute, Open Approach	ICD-10-PCS
0SRD0LZ	Replacement of Left Knee Joint with Unicondylar Synthetic Substitute, Open Approach	ICD-10-PCS
0SRT07Z	Replacement of Right Knee Joint, Femoral Surface with Autologous Tissue Substitute, Open Approach	ICD-10-PCS
0SRT0JZ	Replacement of Right Knee Joint, Femoral Surface with Synthetic Substitute, Open Approach	ICD-10-PCS
0SRT0KZ	Replacement of Right Knee Joint, Femoral Surface with Nonautologous Tissue Substitute, Open Approach	ICD-10-PCS

0SRU07Z	Replacement of Left Knee Joint, Femoral Surface with Autologous Tissue Substitute, Open Approach	ICD-10-PCS
0SRU0JZ	Replacement of Left Knee Joint, Femoral Surface with Synthetic Substitute, Open Approach	ICD-10-PCS
0SRU0KZ	Replacement of Left Knee Joint, Femoral Surface with Nonautologous Tissue Substitute, Open Approach	ICD-10-PCS
0SRV07Z	Replacement of Right Knee Joint, Tibial Surface with Autologous Tissue Substitute, Open Approach	ICD-10-PCS
0SRV0JZ	Replacement of Right Knee Joint, Tibial Surface with Synthetic Substitute, Open Approach	ICD-10-PCS
0SRV0KZ	Replacement of Right Knee Joint, Tibial Surface with Nonautologous Tissue Substitute, Open Approach	ICD-10-PCS
0SRW07Z	Replacement of Left Knee Joint, Tibial Surface with Autologous Tissue Substitute, Open Approach	ICD-10-PCS
0SRW0JZ	Replacement of Left Knee Joint, Tibial Surface with Synthetic Substitute, Open Approach	ICD-10-PCS
0SRW0KZ	Replacement of Left Knee Joint, Tibial Surface with Nonautologous Tissue Substitute, Open Approach	ICD-10-PCS
0SUV09Z	Supplement Right Knee Joint, Tibial Surface with Liner, Open Approach	ICD-10-PCS
0SUW09Z	Supplement Left Knee Joint, Tibial Surface with Liner, Open Approach	ICD-10-PCS
0SWC0JC	Revision of Synthetic Substitute in Right Knee Joint, Patellar Surface, Open Approach	ICD-10-PCS
0SWC0JZ	Revision of Synthetic Substitute in Right Knee Joint, Open Approach	ICD-10-PCS
0SWC3JC	Revision of Synthetic Substitute in Right Knee Joint, Patellar Surface, Percutaneous Approach	ICD-10-PCS
0SWC3JZ	Revision of Synthetic Substitute in Right Knee Joint, Percutaneous Approach	ICD-10-PCS
0SWC4JC	Revision of Synthetic Substitute in Right Knee Joint, Patellar Surface, Percutaneous Endoscopic Approach	ICD-10-PCS
0SWC4JZ	Revision of Synthetic Substitute in Right Knee Joint, Percutaneous Endoscopic Approach	ICD-10-PCS
0SWD0JC	Revision of Synthetic Substitute in Left Knee Joint, Patellar Surface, Open Approach	ICD-10-PCS
0SWD0JZ	Revision of Synthetic Substitute in Left Knee Joint, Open Approach	ICD-10-PCS
0SWD3JC	Revision of Synthetic Substitute in Left Knee Joint, Patellar Surface, Percutaneous Approach	ICD-10-PCS
0SWD3JZ	Revision of Synthetic Substitute in Left Knee Joint, Percutaneous Approach	ICD-10-PCS
0SWD4JC	Revision of Synthetic Substitute in Left Knee Joint, Patellar Surface, Percutaneous Endoscopic Approach	ICD-10-PCS
0SWD4JZ	Revision of Synthetic Substitute in Left Knee Joint, Percutaneous Endoscopic Approach	ICD-10-PCS
0SWT0JZ	Revision of Synthetic Substitute in Right Knee Joint, Femoral Surface, Open Approach	ICD-10-PCS
0SWT3JZ	Revision of Synthetic Substitute in Right Knee Joint, Femoral Surface, Percutaneous Approach	ICD-10-PCS
0SWT4JZ	Revision of Synthetic Substitute in Right Knee Joint, Femoral Surface, Percutaneous Endoscopic Approach	ICD-10-PCS

0SWU0JZ	Revision of Synthetic Substitute in Left Knee Joint, Femoral Surface, Open Approach	ICD-10-PCS
0SWU3JZ	Revision of Synthetic Substitute in Left Knee Joint, Femoral Surface, Percutaneous Approach	ICD-10-PCS
0SWU4JZ	Revision of Synthetic Substitute in Left Knee Joint, Femoral Surface, Percutaneous Endoscopic Approach	ICD-10-PCS
0SWV0JZ	Revision of Synthetic Substitute in Right Knee Joint, Tibial Surface, Open Approach	ICD-10-PCS
0SWV3JZ	Revision of Synthetic Substitute in Right Knee Joint, Tibial Surface, Percutaneous Approach	ICD-10-PCS
0SWV4JZ	Revision of Synthetic Substitute in Right Knee Joint, Tibial Surface, Percutaneous Endoscopic Approach	ICD-10-PCS
0SWW0JZ	Revision of Synthetic Substitute in Left Knee Joint, Tibial Surface, Open Approach	ICD-10-PCS
0SWW3JZ	Revision of Synthetic Substitute in Left Knee Joint, Tibial Surface, Percutaneous Approach	ICD-10-PCS
0SWW4JZ	Revision of Synthetic Substitute in Left Knee Joint, Tibial Surface, Percutaneous Endoscopic Approach	ICD-10-PCS

ICD: *International Classification of Diseases*; ICD-9-CM*: International Classification of Diseases, 9th Revision, Clinical Modification*; ICD-10-CM: *International Classification of Diseases, 10th Revision, Clinical Modification*.

**Codes for Mechanical Heart-Valve Procedure**		
**Code**	**Definition**	**Code Type**
33361	Transcatheter aortic valve replacement (TAVR/TAVI) with prosthetic valve; percutaneous femoral artery approach	CPT
33362	Transcatheter aortic valve replacement (TAVR/TAVI) with prosthetic valve; open femoral artery approach	CPT
33363	Transcatheter aortic valve replacement (TAVR/TAVI) with prosthetic valve; open axillary artery approach	CPT
33364	Transcatheter aortic valve replacement (TAVR/TAVI) with prosthetic valve; open iliac artery approach	CPT
33365	Transcatheter aortic valve replacement (TAVR/TAVI) with prosthetic valve; transaortic approach (eg, median sternotomy, mediastinotomy)	CPT
33366	Transcatheter aortic valve replacement (TAVR/TAVI) with prosthetic valve; transapical exposure (eg, left thoracotomy)	CPT
33367	Transcatheter aortic valve replacement (TAVR/TAVI) with prosthetic valve; cardiopulmonary bypass support with percutaneous peripheral arterial and venous cannulation (eg, femoral vessels) (List separately in addition to code for primary procedure)	CPT

33368	Transcatheter aortic valve replacement (TAVR/TAVI) with prosthetic valve; cardiopulmonary bypass support with open peripheral arterial and venous cannulation (eg, femoral, iliac, axillary vessels) (List separately in addition to code for primary procedure)	CPT
33369	Transcatheter aortic valve replacement (TAVR/TAVI) with prosthetic valve; cardiopulmonary bypass support with central arterial and venous cannulation (eg, aorta, right atrium, pulmonary artery) (List separately in addition to code for primary procedure)	CPT
33405	Replacement, aortic valve, with cardiopulmonary bypass	CPT
33406	Replacement, aortic valve, open, with cardiopulmonary bypass; with allograft valve (freehand)	CPT
33410	Replacement, aortic valve, open, with cardiopulmonary bypass; with stentless tissue valve	CPT
33411	Replacement, aortic valve; with aortic annulus enlargement, noncoronary sinus	CPT
33412	Replacement, aortic valve; with transventricular aortic annulus enlargement (Konno procedure)	CPT
33413	Replacement, aortic valve; by translocation of autologous pulmonary valve with allograft<br>replacement of pulmonary valve (Ross procedure)	CPT
33420	Valvotomy, mitral valve; closed heart	CPT
33422	Valvotomy, mitral valve ; open heart, with cardiopulmonary bypass	CPT
33425	Valvuloplasty, mitral valve, with cardiopulmonary bypass	CPT
33426	Valvuloplasty, mitral valve, with cardiopulmonary bypass	CPT
33427	Valvuloplasty, mitral valve, with cardiopulmonary bypass	CPT
33430	Replacement, mitral valve, with cardiopulmonary bypass	CPT
33465	Replacement, tricuspid valve, with cardiopulmonary bypass	CPT
33475	Replacement, pulmonary valve	CPT
92987	Percutaneous balloon valvuloplasty; mitral valve	CPT
35.20	Open and other replacement of unspecified heart valve	ICD-9-PCS
35.22	Open and other replacement of aortic valve	ICD-9-PCS
35.23	Open and other replacement of mitral valve with tissue graft	ICD-9-PCS
35.24	Open and other replacement of mitral valve	ICD-9-PCS
35.97	Percutaneous mitral valve repair with implant	ICD-9-PCS
02RF0JZ	Replacement of Aortic Valve with Synthetic Substitute, Open Approach	ICD-10-PCS
02RF4JZ	Replacement of Aortic Valve with Synthetic Substitute, Percutaneous Endoscopic Approach	ICD-10-PCS
02RG07Z	Replacement of Mitral Valve with Autologous Tissue Substitute, Open Approach	ICD-10-PCS
02RG08Z	Replacement of Mitral Valve with Zooplastic Tissue, Open Approach	ICD-10-PCS
02RG0JZ	Replacement of Mitral Valve with Synthetic Substitute, Open Approach	ICD-10-PCS
02RG0KZ	Replacement of Mitral Valve with Nonautologous Tissue Substitute, Open Approach	ICD-10-PCS
02RG37Z	Replacement of Mitral Valve with Autologous Tissue Substitute, Percutaneous Approach	ICD-10-PCS
02RG38Z	Replacement of Mitral Valve with Zooplastic Tissue, Percutaneous Approach	ICD-10-PCS
02RG3JZ	Replacement of Mitral Valve with Synthetic Substitute, Percutaneous Approach	ICD-10-PCS
02RG3KZ	Replacement of Mitral Valve with Nonautologous Tissue Substitute, Percutaneous Approach	ICD-10-PCS
02RG47Z	Replacement of Mitral Valve with Autologous Tissue Substitute, Percutaneous Endoscopic Approach	ICD-10-PCS

02RG48Z	Replacement of Mitral Valve with Zooplastic Tissue, Percutaneous Endoscopic Approach	ICD-10-PCS
02RG4JZ	Replacement of Mitral Valve with Synthetic Substitute, Percutaneous Endoscopic Approach	ICD-10-PCS
02RG4KZ	Replacement of Mitral Valve with Nonautologous Tissue Substitute, Percutaneous Endoscopic Approach	ICD-10-PCS
02UG3JZ	Supplement Mitral Valve with Synthetic Substitute, Percutaneous Approach	ICD-10-PCS
V42.2	Transplant; heart valve	ICD-9-CM
V43.3	Heart valve replaced by other means	ICD-9-CM
Z95.2	Presence of prosthetic heart valve	ICD-10-CM
Z95.3	Presence of xenogenic heart valve	ICD-10-CM
Z95.4	Presence of other heart-valve replacement	ICD-10-CM

CPT: Common Procedural Terminology

Data S2. Codes for Quan-Charlson Comorbidity Index, CHA_2DS_2-VASc Score, and HAS-BLED Score.

Comorbidities	ICD-9-CM Codes*	ICD-10-CM Codes*	Quan 2011 Weight**
Myocardial infarction	410.%, 412.%	I21.%, I22.%, I25.2%	0
Congestive heart failure	398.91, 402.01, 402.11, 402.91, 404.01, 404.03, 404.11, 404.13, 404.91, 404.93, 425.4%-425.9%, 428.%	I09.9%, I11.0%, I13.0%, I13.2%, I25.5%, I42.0%, I42.5%–I42.9%, I43.%, I50.%, P29.0%	2
Peripheral vascular disease	093.0%, 437.3%, 440.%, 441.%, 443.1%-443.9%, 447.1% 557.1%, 557.9%, V43.4%	I70.%, I71.%, I73.1%, I73.8%, I73.9%, I77.1%, I79.0%, I79.2%, K55.1%, K55.8%, K55.9%, Z95.8%, Z95.9%	0
Cerebrovascular disease	362.34, 430.% – 438.%	G45.%, G46.%, H34.0%, I60.%–I69.%	0
Dementia	290.%, 294.1%, 331.2%	F00.%–F03.%, F05.1, G30.%, G31.1%	2
Chronic Pulmonary Disease	416.8%, 416.9%, 490.%-505.%, 506.4%, 508.1%, 508.8%	I27.8%, I27.9%, J40.%–J47.%, %, J68.4%, J70.1%, J70.3%	1
Connective tissue/rheumatic disease	446.5%, 710.0% – 710.4%, 714.0% – 714.2%, 714.8%, 725.%	M05.%, M06.%, M31.5%, 2.%–M34.%, M35.1%, M35.3%, M36.0%	1
Peptic ulcer disease	531.% – 534.%	K25.%–K28.%	0
Mild liver disease:	070.22, 070.23, 070.32, 070.33, 070.44, 070.54, 070.6%, 070.9%, 570.%, 571.%, 573.3%, 573.4%, 573.8%, 573.9%, V42.7%	B18.%, K70.0%–K70.3%, K70.9%, K71.3%–K71.5%, K71.7%, K73.%, K74.%, K76.0%, K76.2%–K76.4%, K76.8%, K76.9%, Z94.4%	2
Diabetes without chronic complications/mild to moderate	250.0% – 250.3%; 250.8%, 250.9%	E10.0%, E10.1%, E10.6%, E10.8%, E10.9%, E11.0%, E11.1%, E11.6%, E11.8%, E11.9%, E12.0%, E12.1%, E12.6%, E12.8%, E12.9%, E13.0%, E13.1%, E13.6%, E13.8%, E13.9%, E14.0%, E14.1%, E14.6%, E14.8%, E14.9%	0
Paraplegia and hemiplegia	334.1%, 342.%, 343.%, 344.0%-344.6%, 344.9%	G04.1%, G11.4%, G80.1%, G80.2%, G81.%, G82.%, G83.0%–G83.4%, G83.9%	2
Renal disease	403.01, 403.11, 403.91, 404.02, 404.03, 404.12, 404.13, 404.92, 404.93, 582.0% – 583.7%, 585.%, 586.%, 588.0%, V42.0%, V45.1%, V56.%	I12.0%, I13.1%, N03.2%–N03.7%, N05.2%–N05.7%, N18.%, N19.%, N25.0%, Z49.0%–Z49.2%, Z94.0%, Z99.2%	1
Diabetes with chronic complications	250.4% – 250.7%	E10.2%–E10.5%, E10.7%, E11.2%–E11.5%, E11.7%, E12.2%–E12.5%, E12.7%,	1

		E13.2%–E13.5%, E13.7%, E14.2%–E14.5%, E14.7%	
Any malignancy, including lymphoma and leukemia, except malignant neoplasm of skin	140.% – 172.%, 174.% – 195.8%, 200.% – 208.%, 238.6%	C00.%–C26.%, C30.%–C34.%, C37.%–C41.%, C43.%, C45.%–C58.%, C60.%–C76.%, C81.%–C85.%, C88.%, C90.%–C97.%	2
Moderate or severe liver disease	456.0%-456.2%, 572.2%-572.8%	I85.0%, I85.9%, I86.4%, I98.2%, K70.4%, K71.1%, K72.1%, K72.9%, K76.5%, K76.6%, K76.7%	4
Metastatic solid tumor	196.% – 199.%	C77.%–C80.%	6
AIDS/HIV	042.%, 043.%, 044.%	B20.%–B22.%, B24.%	4

The table above outlines the updated QCI index (*Quan* et al 2011). To quantify comorbidity, the QCI score is computed by adding the weights that are assigned to the specific diagnoses. Each diagnosis is only counted once. The minimum possible score is 0 and the maximum possible score is 24.

Reference:

* Quan et al. Coding algorithms for defining comorbidities in ICD-9-CM and ICD-10 administrative data. *Med Care*. 2005 Nov;43(11):1130-1139.

** Quan et al. Updating and Validating the Charlson Comorbidity Index and Score for Risk Adjustment in Hospital Discharge Abstracts Using Data From Six Countries. *Am J Epidemiol*. 2011. Mar 15;173(6):676-682.

Scoring Algorithm for CHA_2DS_2-VASc Score

	**Condition (Original)**	**Condition (Modified)**	**Points**
**C**	Congestive heart failure (or Left ventricular systolic dysfunction)	≥1 diagnosis for congestive heart failure	1
**H**	Hypertension: blood pressure consistently above 140/90 mmHg (or treated hypertension on medication)	≥1 diagnosis for hypertension; AND ≥1 prescription fill for antihypertensive or diuretic medications	1
**A_2**	Age ≥75 years	Age ≥75 years as of the index date	2
**D**	Diabetes Mellitus	≥1 diagnosis for diabetes mellitus; OR ≥1 prescription fill for antidiabetic medications	1
**S_2**	Prior Stroke or TIA or thromboembolism	≥1 diagnosis for stroke, or TIA, or thromboembolism	2
**V**	Vascular disease (e.g. peripheral artery disease, myocardial infarction, aortic plaque)	≥1 diagnosis for vascular disease	1
**A**	Age 65–74 years	Age 65–74 years as of the index date	1
**Sc**	Female sex	Female sex	1

Codes for Diseases Included in CHA_2DS_2-VASc Score

Disease of Interest	ICD-9-CM	ICD-10-CM
Congestive heart failure	428.%	I50.%
Hypertension	401.% - 405.%	I10.% - I16.%
Diabetes mellitus	250.%	E10.% - E13.%
Prior stroke or TIA or thromboembolism	431.% - 435.%	I61.% - I66.%, G45.%
Vascular disease and myocardial infarction	440.% - 449.%, 410.%, 412.%	I70.% - I79.%, I21.%, I22.%, I25.2%

Codes for Medications Included in CHA_2DS_2-VASc Score

Medication of Interest	GPI	HCPCS
Antihypertensive medications	36%	, J0360, S0139, J1730, J2670
Diuretics	37%, 369918%, 369920%, 369940%, 369945%, 369950%, 369955%, 369960%, 369968%, 369990%	S0171, J1940, J3265, J2150, J7665, J3350, J1205
Antidiabetic medications	27%	317, E0784, S5550-S5571, G9147, S9353

Scoring algorithm for HAS-BLED score*

Abbreviation	Condition (Original)	Condition (Modified)	Points
H	Hypertension	≥1 diagnosis for hypertension	1
A	Abnormal renal and liver function (1 point each)	≥1 diagnosis for renal disease; OR ≥1 diagnosis for hepatic-liver disease	1 or 2
S	Stroke	≥1 diagnosis for stroke	2
B	Bleeding	≥1 diagnosis for previous bleeding	1
L	Labile INRs	N/A - Omitted	2
E	Elderly (>65 years)	Age >65 years as of the index date	1
D	Drugs or alcohol concomitantly (1 point each)	≥1 diagnosis for ethanol abuse; OR ≥1 prescription fill for NSAIDs or antiplatelet	1 or 2

*PISTERS et al. A novel user-friendly score (HAS-BLED) to assess 1-year risk of major bleeding in patients with AF. *CHEST* 2010. 138(5).

Codes for diseases involved in HAS-BLED score

Predictor	Description	ICD-9-CM	ICD-10-CM
**Hypertension**	Essential hypertension	401.x	I10.%, I16.%
	Hypertensive heart disease	402.x	I11.%
	Hypertensive chronic kidney disease	403.x	I12.%
	Hypertensive heart and chronic kidney disease	404.x	I13.%
	Secondary hypertension	405.x	I15.%
**Renal disease**	Diabetes with renal manifestations	250.4x	E10.21%, E10.29%, E10.65%, E11.21%, E11.29%, E11.65%
	Hypertensive chronic kidney disease	403.xx	I12.%
	Hypertensive heart and chronic kidney disease	404.xx	I13.%
	Malignant, renovascular secondary hypertension	405.01	I15.0%
	Benign, renovascular secondary hypertension	405.11	I15.0%
	Unspecified, renovascular secondary hypertension	405.91	I15.0%
	Acute kidney failure	584.x	N17.%
	Chronic kidney disease	585.x	N18.%
	Renal failure, unspecified	586.x	N19%
	Disorders resulting from impaired renal function	588.xx	N25.%
	Renal agenesis and dysgenesis	753.0x	Q60.0%, Q60.1%, Q60.2%, Q60.3%, Q60.4%, Q60.5%
	Cystic kidney disease	753.1x	Q61.%
	Proteinuria	791.0x	R80.%
	Postsurgical renal dialysis status	V45.1x	Z99.2%, Z91.15%
	Encounter for dialysis and dialysis catheter care	V56.xx	Z49.%
**Hepatic-liver disease**	Acute and subacute necrosis of liver	570.x	K76.2%, K72.0%

	Chronic liver disease and cirrhosis	571.xx	K70.%, K71.7%, K73.%, K74.%, K75.4%, K75.81%, K76.0%, K76.89%, K76.9%
	Liver abscess and sequelae of chronic liver disease	572.x	K72.01%, K72.1%, K72.9%, K75.0%, K75.1%, K76.6%, K76.7%
	Other disorders of liver	573.x	K71.%, K75.2%, K75.3%, K75.89%, K75.9%, K76.1%, K76.3%, K76.4%, K76.5%, K76.8%, K76.9%, K77.%
	Congenital cystic disease of liver	751.62	Q44.6%
	Glycogenosis	271.0x	E74.0%, E74.4%
**Stroke**	Ischemic and/or hemorrhagic stroke		
	Subarachnoid hemorrhage	430.x	I60.%
	Intracerebral hemorrhage	431.x	I61.%
	Other and unspecified intracranial hemorrhage	432.x	I62.%
	Occlusion and stenosis of precerebral arteries	433.x	I65.%, I63.0% - I63.2%
	Occlusion of cerebral arteries	434.x	I66.%, I63.3% - I63.9%
	Acute, but ill-defined, cerebrovascular disease	436.x	I67.89%
	Other generalized ischemic cerebrovascular disease	437.1x	I67.81%, I67.82%, I67.89%
	Cerebral aneurysm, nonruptured	437.3x	I67.1%
	Iatrogenic cerebrovascular infarction or hemorrhage	997.02	I97.81%, I97.82%, G97.3%
	Transient ischemic attack		
	Transient cerebral ischemia	435.x	G45.0% - G45.2%, G45.8%, G45.9%, I67.84%
**Previous bleeding**	Other disease of paricardium	423.x	I31.%
	Hemoparicardium	423.0x	I31.2%
	Subarachnoid hemorrhage	430.x	I60.%
	Intracerebral hemorrhage	431.x	I61.%
	Other and unspecified intracranial hemorrhage	432.x	I62.%
	Internal hemorrhoid with other complications – bleeding	455.2x	K64.%

External hemorrhoids with other complication	455.5x	K64.%
Unspecified hemorrhoids with other complication	455.8x	K64.%
Esophageal varices with bleeding	456.0x	I85.01%
Esophageal varices in disease classified elsewhere, with bleeding	456.20	I85.11%
Hemorrhage, unspecified	459.0x	R58.%
Gastroesophageal laceration-hemorrhage syndrome	530.7x	K22.6%
Esophageal hemorrhage	530.82	K22.8%
Gastric ulcer, acute with hemorrhage, without obstruction	531.00	K25.0%
Gastric ulcer, acute with hemorrhage, with obstruction	531.01	K25.0%
Gastric ulcer, acute with hemorrhage and perforation, without obstruction	531.20	K25.2%
Gastric ulcer, acute with hemorrhage and perforation, with obstruction	531.21	K25.2%
Gastric ulcer, chronic or unspecified with hemorrhage, without obstruction	531.40	K25.4%
Gastric ulcer, chronic or unspecified with hemorrhage, with obstruction	531.41	K25.4%
Gastric ulcer, chronic or unspecified with hemorrhage and perforation, without obstruction	531.60	K25.6%
Gastric ulcer, chronic or unspecified with hemorrhage and perforation, with obstruction	531.61	K25.6%

Duodenal ulcer, acute with hemorrhage, without obstruction	532.00	K26.0%
Duodenal ulcer, acute with hemorrhage, with obstruction	532.01	K26.0%
Duodenal ulcer, acute with hemorrhage and perforation, without obstruction	532.20	K26.2%
Duodenal ulcer, acute with hemorrhage and perforation, with obstruction	532.21	K26.2%
Duodenal ulcer, chronic or unspecified with hemorrhage, without obstruction	532.40	K26.4%
Duodenal ulcer, chronic or unspecified with hemorrhage, with obstruction	532.41	K26.4%
Duodenal ulcer, chronic or unspecified with hemorrhage and perforation, without obstruction	532.60	K26.6%
Duodenal ulcer, chronic or unspecified with hemorrhage and perforation, with obstruction	532.61	K26.6%
Peptic ulcer, acute with hemorrhage, without obstruction	533.00	K27.0%
Peptic ulcer, acute with hemorrhage, with obstruction	533.01	K27.0%
Peptic ulcer, acute with hemorrhage and perforation, without obstruction	533.20	K27.2%
Peptic ulcer, acute with hemorrhage and perforation, with obstruction	533.21	K27.2%
Peptic ulcer, chronic or unspecified with hemorrhage, without obstruction	533.40	K27.4%
Peptic ulcer, chronic or unspecified with hemorrhage, with obstruction	533.41	K27.4%
Peptic ulcer, chronic or unspecified with hemorrhage and perforation, without obstruction	533.60	K27.6%
Peptic ulcer, chronic or unspecified with hemorrhage and perforation, with obstruction	533.61	K27.6%

Gastrojejunal ulcer, acute with hemorrhage, without obstruction	534.00	K28.0%
Gastrojejunal ulcer, acute with hemorrhage, with obstruction	534.01	K28.0%
Gastrojejunal ulcer, acute with hemorrhage and perforation, without obstruction	534.20	K28.2%
Gastrojejunal ulcer, acute with hemorrhage and perforation, with obstruction	534.21	K28.2%
Gastrojejunal ulcer, chronic or unspecified with hemorrhage, without obstruction	534.40	K28.4%
Gastrojejunal ulcer, chronic or unspecified with hemorrhage, with obstruction	534.41	K28.4%
Gastrojejunal ulcer, chronic or unspecified with hemorrhage and perforation, without obstruction	534.60	K28.6%
Gastrojejunal ulcer, chronic or unspecified with hemorrhage and perforation, with obstruction	534.61	K28.6%
Acute gastritis, with hemorrhage	535.01	K29.01%
Atrophic gastritis, with hemorrhage	535.11	K29.41%, K29.51%
Gastric mucosal hypertrophy, with hemorrhage	535.21	K29.61%
Alcoholic gastritis, with hemorrhage	535.31	K29.21%
Other specified gastritis, with hemorrhage	535.41	K29.61%
Unspecified gastritis and gastroduodenitis, with hemorrhage	535.51	K29.71%, K29.91%
Duodenitis, with hemorrhage	535.61	K29.81%
Angiodysplasia of stomach and duodenum with hemorrhage	537.83	K31.811
Diverticulosis of small intestine with hemorrhage	562.02	K57.11%
Diverticulitis of small intestine with hemorrhage	562.03	K57.13%
Diverticulosis of colon with hemorrhage	562.12	K57.31%
Diverticulitis of colon with hemorrhage	562.13	K57.33%
Hemoperitoneum	568.81	K66.1%
Hemorrhage of rectum and anus	569.3x	K62.5%

	Angiodysplasia of intestine with hemorrhage	569.85	K55.21%
	Hematemesis	578.0x	K92.0%
	Blood in stool	578.1x	K92.1%
	Hemorrhage of gastrointestinal tract, unspecified	578.9x	K92.2%
	Vascular disorders of kidney	593.81	N28.0%
	Hemorrhage into bladder wall	596.7x	N32.89%
	Hematuria	599.7x	R31.%
	Excessive or frequent menstruation	626.2x	N92.0%
	Metrorrhagia	626.6x	N92.1%
	Hemarthrosis	719.1x	M25.0%
	Epistaxis	784.7x	R04.0%
	Hemorrhage from throat	784.8x	R04.1%
	Hemoptysis	786.3x	R04.2%, R04.8%, R04.9%
	Subarachnoid hemorrhage following injury without mention of open intracranial wound	852.0x	S06.6X%
	Subdural hemorrhage following injury without mention of open intracranial wound	852.2x	S06.5X%
	Extradural hemorrhage following injury without mention of open intracranial wound	852.4x	S06.4X%
	Other and unspecified intracranial hemorrhage following injury without mention of open intracranial wound	853.0x	S06.36%A
**Ethanol abuse**	Alcohol-related disorders	291.xx, 303.xx, 305.0x	F10.%
	Alcoholic fatty liver	571.0x	K70.0%
	Acute alcoholic hepatitis	571.1x	K70.1%
	Alcoholic cirrhosis of liver	571.2x	K70.3%
	Alcoholic liver damage, unspecified	571.3x	K70.4%
	Alcoholic gastritis	535.3x	K29.2%
	Alcohol affecting fetus or newborn via placenta or breast milk	760.71	P04.3%, Q86.0%
	Toxic effect of ethyl alcohol	980.0x	T51.0%

ICD-9-CM: *International Classification of Diseases, 9th Revision, Clinical Modification*; ICD-10-CM: *International Classification of Diseases, 10th Revision, Clinical Modification*.

Codes for Medications Involved in HAS-BLED Score

Predictor	Description	GPI Code	HCPCS Code
**NSAIDs**	Nonsteroidal Anti-inflammatory Agents (NSAIDs)	6610%	J1130, J1741, J1885
**Antiplatelet**	Platelet Aggregation Inhibitors	8515%	J1245, J0130, J1327, J3246, C9460

GPI: Generic Product Identifier.

<u>References:</u>

Pisters R, et al. A novel user-friendly score (HAS-BLED) to assess 1-year risk of major bleeding in patients with AF. *CHEST*, 2010;138(5)

Lamberts M, et al. Antithrombotic treatment in patients with heart failure and associated atrial fibrillation and vascular disease: A nationwide cohort study. *J Am Coll Cardiol*, 2014;63(24):2689-2698.

Data S3. Codes for Prior Medications of Interest

Drug class/subclass	GPI code start with	HCPCS Code
Anti-hyperlipidemics	39%	N/A
Anti-hypertensives	33%, 36%, 37%	J0210, J2760, J0360, S0139, J1730, J2670, J1800
Anti-platelets	8515%	J1245, J0130, J1327, J3246, C9460
Beta-blockers	33%	
Calcium channel blockers	34%	
Digoxin	31200010%	
Anti-arrhythmias	35100010%, 35100030%, 35300010%, 35300050%, 35400005%, 35400028%, 33100045%,	J1742, J1160, J2001, J0153, J0282

Table S1. Demographic and baseline characteristics after matching for patients with 1–4 concurrent medications.

	After matching		
**Characteristic**	**Rivaroxaban (n=7593)**	**Warfarin (n=7593)**	**Standardized mean difference***
Age, years, median (IQR)	62 (56-70)	62 (56-70)	0.8%
Sex, n (%)			
Male	5146 (67.8)	5146 (67.8)	0.0%
Female	2447 (32.2)	2447 (32.2)	0.0%
Insurance type, n (%)			
Commercial	4868 (64.1)	4868 (64.1)	0.0%
Medicare	2725 (35.9)	2725 (35.9)	0.0%
Baseline risk scores, median (IQR)			
QCI	0 (0-2)	1 (0-2)	5.6%
CHA_2DS_2-VASC	2 (1-3)	2 (1-3)	3.9%
HAS-BLED	2 (1-3)	2 (1-3)	2.2%
Baseline concurrent medications, n (%)			
1-4	7593 (100)	7593 (100)	-
5-9	-	-	-
≥10	-	-	-
Baseline obesity class, n (%)			
Class I (BMI 30-34 kg/m^2)	4410 (58.1)	4423 (58.3)	0.3%
Class II (BMI 35-39 kg/m^2)	1014 (13.4)	1055 (13.9)	1.6%

Class III (BMI ≥40.0 kg/m^2)	2169 (28.6)	2115 (27.9)	1.6%
Most common baseline comorbidities (>10%†), n (%)			
Hypertension	6010 (79.2)	5981 (78.8)	0.9%
Hyperlipidemia	4269 (56.2)	4151 (54.7)	3.1%
Diabetes without chronic complications	2140 (28.2)	2233 (29.4)	2.7%
Congestive heart failure	1832 (24.1)	2015 (26.5)	5.5%
Osteoarthritis	1698 (22.4)	1659 (21.8)	1.2%
Chronic pulmonary disease	1469 (19.3)	1564 (20.6)	3.1%
Cancer	1684 (22.2)	1659 (21.8)	0.8%
Thyroid disease	1192 (15.7)	1075 (14.2)	4.3%
Coronary artery disease	751 (9.9)	904 (11.9)	6.5%
Chronic obstructive pulmonary disease	757 (10.0)	807 (10.6)	2.2%
Diabetes with complications	505 (6.7)	594 (7.8)	4.5%
Anemia	848 (11.2)	988 (13.0)	5.7%
Peripheral vascular disease	789 (10.4)	861 (11.3)	3.0%
Cerebrovascular disease	793 (10.4)	891 (11.7)	4.1%
Asthma	583 (7.7)	594 (7.8)	0.5%
Depression	512 (6.7)	534 (7.0)	1.1%
Anxiety	513 (6.8)	445 (5.9)	3.7%
Renal disease	552 (7.3)	647 (8.5)	4.6%
Myocardial infarction	486 (6.4)	535 (7.0)	2.6%

Baseline procedures, n (%)			
Gastric bypass surgery	32 (0.4)	31 (0.4)	0.2%
Catheter ablation	369 (4.9)	335 (4.4)	2.1%
Coronary bypass graft	118 (1.6)	169 (2.2)	4.9%
Percutaneous coronary intervention	138 (1.8)	145 (1.9)	0.7%
Cardioversion	141 (1.9)	121 (1.6)	2.0%
Baseline medication use, n (%)			
Nonoral anticoagulants	723 (9.5)	785 (10.3)	2.7%
Antihyperlipidemics	275 (3.6)	295 (3.9)	1.4%
Antihypertensives	6404 (84.3)	6438 (84.8)	1.2%
Antiplatelet agents	418 (5.5)	416 (5.5)	0.1%
Antiarrhythmia agents	5625 (74.1)	5669 (74.7)	1.3%
Beta-blockers	4322 (56.9)	4402 (58.0)	2.1%
Calcium channel blockers	2074 (27.3)	2092 (27.6)	0.5%
Digoxin	420 (5.5)	492 (6.5)	4.0%
Baseline all-cause HRU counts, median (IQR)			
Inpatient hospitalization	1 (0-1)	1 (0-1)	2.0%
ER visit	0 (0-0)	0 (0-0)	1.6%
Office visit	7 (4-11)	7 (4-12)	5.3%
Outpatient visit	28 (14-54)	32 (17-57)	5.1%
Pharmacy fill	20 (12-30)	21 (13-31)	4.2%
SNF/long-term care (yes), n (%)	250 (3.3)	322 (4.2)	5.0%

Baseline costs ($), median (IQR)			
Inpatient hospitalization	400.60 (0-15,467)	22.01 (0-17,403)	4.9%
ER visit	0 (0-0)	0 (0-0)	0.5%
Office visit	819.25 (460-1364)	835.12 (458-1396)	2.9%
Outpatient visit	2556.35 (847-6996)	2546.77 (834-6940)	2.0%
Pharmacy fill	1099.90 (377-2845)	1070.50 (363-2779)	2.0%
SNF visit	0 (0-0)	0 (0-0)	3.0%

BMI body mass index, *ER* emergency room, *HRU* healthcare resource utilization, *QCI* Quan-Charlson comorbidity index, *SD* standard deviation, *SNF* skilled nursing facility.

*A standardized mean difference <10% was considered a negligible imbalance.
[†]10% or more in either treatment cohort before matching.

Table S2. Demographic and baseline characteristics after matching for patients with 5–9 concurrent medications.

	After matching		
**Characteristic**	**Rivaroxaban (n=8626)**	**Warfarin (n=8626)**	**Standardized mean difference***
Age, years, median (IQR)	66 (60-74)	66 (60-74)	0.5%
Sex, n (%)			
Male	5357 (62.1)	5357 (62.1)	0.0%
Female	3269 (37.9)	3269 (37.9)	0.0%
Insurance type, n (%)			
Commercial	4219 (48.9)	4219 (48.9)	0.0%
Medicare	4407 (51.1)	4407 (51.1)	0.0%
Baseline risk scores, median (IQR)			
QCI	1 (0-3)	2 (0-3)	7.1%
CHA_2DS_2-VASC	3 (2-4)	3 (2-5)	5.9%
HAS-BLED	2 (1-3)	2 (1-3)	3.7%
Baseline concurrent medications, n (%)			
1-4	-	-	-
5-9	8626 (100)	8626 (100)	-
≥10	-	-	-
Baseline obesity class, n (%)			
Class I (BMI 30-34 kg/m^2)	4342 (50.3)	4232 (49.1)	2.6%
Class II (BMI 35-39 kg/m^2)	1287 (14.9)	1318 (15.3)	1.0%

Class III (BMI ≥40.0 kg/m^2)	2997 (34.7)	3076 (35.7)	1.9%
Most common baseline comorbidities (>10%[†]), n (%)			
Hypertension	7609 (88.2)	7470 (86.6)	4.9%
Hyperlipidemia	5932 (68.8)	5692 (66.0)	5.9%
Diabetes without chronic complications	4306 (49.9)	4543 (52.7)	5.5%
Congestive heart failure	2870 (33.3)	3136 (36.4)	6.5%
Osteoarthritis	2367 (27.4)	2284 (26.5)	2.2%
Chronic pulmonary disease	2462 (28.5)	2597 (30.1)	3.4%
Cancer	2579 (29.9)	2537 (29.4)	1.1%
Thyroid disease	1864 (21.6)	1755 (20.3)	3.1%
Coronary artery disease	1415 (16.4)	1681 (19.5)	8.0%
Chronic obstructive pulmonary disease	1415 (16.4)	1597 (18.5)	5.6%
Diabetes with complications	1430 (16.6)	1595 (18.5)	5.0%
Anemia	1366 (15.8)	1658 (19.2)	8.9%
Peripheral vascular disease	1356 (15.7)	1522 (17.6)	5.2%
Cerebrovascular disease	1347 (15.6)	1460 (16.9)	3.5%
Asthma	1046 (12.1)	995 (11.5)	1.8%
Depression	892 (10.3)	925 (10.7)	1.2%
Anxiety	778 (9.0)	692 (8.0)	3.6%
Renal disease	1197 (13.9)	1494 (17.3)	9.5%
Myocardial infarction	860 (10.0)	945 (11.0)	3.2%

Baseline procedures, n (%)			
Gastric bypass surgery	38 (0.4)	44 (0.5)	1.0%
Catheter ablation	319 (3.7)	309 (3.6)	0.6%
Coronary bypass graft	189 (2.2)	279 (3.2)	6.4%
Percutaneous coronary intervention	259 (3.0)	280 (3.2)	1.4%
Cardioversion	177 (2.1)	158 (1.8)	1.6%
Baseline medication use, n (%)			
Nonoral anticoagulants	980 (11.4)	1015 (11.8)	1.3%
Antihyperlipidemics	850 (9.9)	976 (11.3)	4.8%
Antihypertensives	8357 (96.9)	8338 (96.7)	1.3%
Antiplatelet agents	1154 (13.4)	1144 (13.3)	0.3%
Antiarrhythmia agents	7644 (88.6)	7638 (88.5)	0.2%
Beta-blockers	6378 (73.9)	6375 (73.9)	0.1%
Calcium channel blockers	3525 (40.9)	3512 (40.7)	0.3%
Digoxin	956 (11.1)	1074 (12.5)	4.2%
Baseline all-cause HRU counts, median (IQR)			
Inpatient hospitalization	1 (0-1)	1 (0-1)	3.2%
ER visit	0 (0-0)	0 (0-1)	2.1%
Office visit	10 (6-16)	10 (6-16)	6.6%
Outpatient visit	41 (23-72)	46 (25-77)	7.7%
Pharmacy fill	39 (28-55)	40 (30-56)	4.5%
SNF/long-term care (yes), n (%)	386 (4.5)	451 (5.2)	3.5%

Baseline costs ($), median (IQR)			
Inpatient hospitalization	3679.50 (0-20,342)	3968.24 (0-24,041)	8.6%
ER visit	0 (0-0)	0 (0-14)	0.8%
Office visit	1153.21 (701-1803)	1182.58 (699-1860)	4.0%
Outpatient visit	3935.79 (1521-10,040)	4073.80 (1563-10,522)	4.8%
Pharmacy fill	3427.51 (1452-6390)	3420.99 (1450-6439)	2.4%
SNF visit	0 (0-0)	0 (0-0)	1.5%

BMI body mass index, *ER* emergency room, *HRU* healthcare resource utilization, *QCI* Quan-Charlson comorbidity index, *SD* standard deviation, *SNF* skilled nursing facility.

*A standardized mean difference <10% was considered a negligible imbalance.

†10% or more in either treatment cohort before matching.

Table S3. Demographic and baseline characteristics after matching for patients with 10+ concurrent medications.

	After matching		
**Characteristic**	**Rivaroxaban (n=2992)**	**Warfarin (n=2992)**	**Standardized mean difference***
Age, years, median (IQR)	68 (62-75)	68 (62-76)	0.4%
Sex, n (%)			
Male	1747 (58.4)	1747 (58.4)	0.0%
Female	1245 (41.6)	1245 (41.6)	0.0%
Insurance type, n (%)			
Commercial	1224 (40.9)	1224 (40.9)	0.0%
Medicare	1768 (59.1)	1768 (59.1)	0.0%
Baseline risk scores, median (IQR)			
QCI	2 (1-4)	3 (1-4)	6.7%
CHA_2DS_2-VASC	4 (3-5)	4 (3-5)	4.3%
HAS-BLED	3 (2-4)	3 (2-4)	3.0%
Baseline concurrent medications, n (%)			
1-4	-	-	-
5-9	-	-	-
≥10	2992 (100)	2992 (100)	-
Baseline obesity class, n (%)			
Class I (BMI 30-34 kg/m^2)	1099 (36.7)	1086 (36.3)	0.9%
Class II (BMI 35-39 kg/m^2)	494 (16.5)	463 (15.5)	2.8%

Class III (BMI ≥40.0 kg/m^2)	1399 (46.8)	1443 (48.2)	2.9%
Most common baseline comorbidities (>10%†), n (%)			
Hypertension	2708 (90.5)	2675 (89.4)	3.7%
Hyperlipidemia	2154 (72.0)	2109 (70.5)	3.3%
Diabetes without chronic complications	2170 (72.5)	2194 (73.3)	1.8%
Congestive heart failure	1425 (47.6)	1486 (49.7)	4.1%
Osteoarthritis	1021 (34.1)	980 (32.8)	2.9%
Chronic pulmonary disease	1351 (45.2)	1346 (45.0)	0.3%
Cancer	1124 (37.6)	1108 (37.0)	1.1%
Thyroid disease	796 (26.6)	777 (26.0)	1.4%
Coronary artery disease	761 (25.4)	840 (28.1)	6.0%
Chronic obstructive pulmonary disease	949 (31.7)	992 (33.2)	3.1%
Diabetes with complications	984 (32.9)	1061 (35.5)	5.4%
Anemia	739 (24.7)	807 (27.0)	5.2%
Peripheral vascular disease	684 (22.9)	727 (24.3)	3.4%
Cerebrovascular disease	661 (22.1)	709 (23.7)	3.8%
Asthma	611 (20.4)	506 (16.9)	9.0%
Depression	518 (17.3)	494 (16.5)	2.1%
Anxiety	437 (14.6)	362 (12.1)	7.4%
Renal disease	721 (24.1)	863 (28.8)	10.8%
Myocardial infarction	433 (14.5)	485 (16.2)	4.8%

Baseline procedures, n (%)			
Gastric bypass surgery	7 (0.2)	5 (0.2)	1.5%
Catheter ablation	100 (3.3)	93 (3.1)	1.3%
Coronary bypass graft	90 (3.0)	111 (3.7)	3.9%
Percutaneous coronary intervention	156 (5.2)	172 (5.7)	2.4%
Cardioversion	61 (2.0)	60 (2.0)	0.2%
Baseline medication use, n (%)			
Nonoral anticoagulants	420 (14.0)	471 (15.7)	4.8%
Antihyperlipidemics	619 (20.7)	675 (22.6)	4.5%
Antihypertensives	2956 (98.8)	2945 (98.4)	3.1%
Antiplatelet agents	768 (25.7)	753 (25.2)	1.2%
Antiarrhythmia agents	2783 (93.0)	2805 (93.8)	3.0%
Beta-blockers	2399 (80.2)	2421 (80.9)	1.9%
Calcium channel blockers	1501 (50.2)	1497 (50.0)	0.3%
Digoxin	470 (15.7)	497 (16.6)	2.5%
Baseline all-cause HRU counts, median (IQR)			
Inpatient hospitalization	1 (0-2)	1 (0-2)	2.2%
ER visit	0 (0-1)	0 (0-1)	4.3%
Office visit	14 (9-21)	15 (9-22)	4.9%
Outpatient visit	62 (35-105)	67 (39-108)	6.9%
Pharmacy fill	66 (50-95)	67 (52-94.5)	2.3%
SNF/long-term care (yes), n (%)	238 (8.0)	281 (9.4)	5.1%

Baseline costs ($), median (IQR)			
Inpatient hospitalization	10,389.26 (0-33,046)	11,100.19 (0-38,121)	6.7%
ER visit	0 (0-616)	0 (0-635.35)	1.6%
Office visit	1617.41 (1011-2497)	1665.09 (1005-2610)	4.1%
Outpatient visit	6545.53 (2773-15,376)	7011.87 (2920-16,632)	6.0%
Pharmacy fill	8229.91 (4676-13,737)	8358.00 (4658-13,921)	1.0%
SNF visit	0 (0-0)	0 (0-0)	3.2%

BMI body mass index, *ER* emergency room, *HRU* healthcare resource utilization, *QCI* Quan-Charlson comorbidity index, *SD* standard deviation, *SNF* skilled nursing facility.

*A standardized mean difference <10% was considered a negligible imbalance.

†10% or more in either treatment cohort before matching.

Table S4. Demographic and baseline characteristics after matching for patients with BMI of 30–34 kg/m^2.

	After matching		
**Characteristic**	**Rivaroxaban (n=9858)**	**Warfarin (n=9858)**	**Standardized mean difference***
Age, years, median (IQR)	67 (60-75)	67 (60-75)	1.1%
Sex, n (%)			
Male	6980 (70.8)	6980 (70.8)	0.0%
Female	2878 (29.2)	2878 (29.2)	0.0%
Insurance type, n (%)			
Commercial	4486 (45.5)	4486 (45.5)	0.0%
Medicare	5372 (54.5)	5372 (54.5)	0.0%
Baseline risk scores, median (IQR)			
QCI	1 (0-2)	1 (0-3)	7.4%
CHA_2DS_2-VASC	3 (1-4)	3 (1-4)	4.8%
HAS-BLED	2 (1-3)	2 (1-3)	3.4%
Baseline concurrent medications, n (%)			
1-4	4477 (45.4)	4488 (45.5)	0.2%
5-9	4261 (43.2)	4217 (42.8)	0.9%
≥10	1120 (11.4)	1153 (11.7)	1.0%
Baseline obesity class, n (%)			
Class I (BMI 30-34 kg/m^2)	9858 (100)	9858 (100)	-
Class II (BMI 35-39 kg/m^2)	-	-	-

Class III (BMI ≥40.0 kg/m^2)	-	-	-
Most common baseline comorbidities (>10%[†]), n (%)			
Hypertension	8110 (82.3)	7995 (81.1)	3.0%
Hyperlipidemia	6343 (64.3)	6204 (62.9)	2.9%
Diabetes without chronic complications	3557 (36.1)	3723 (37.8)	3.5%
Congestive heart failure	2646 (26.8)	2960 (30.0)	7.1%
Osteoarthritis	2271 (23.0)	2214 (22.5)	1.4%
Chronic pulmonary disease	2244 (22.8)	2340 (23.7)	2.3%
Cancer	2853 (28.9)	2776 (28.2)	1.7%
Thyroid disease	1767 (17.9)	1645 (16.7)	3.3%
Coronary artery disease	1541 (15.6)	1792 (18.2)	6.8%
Chronic obstructive pulmonary disease	1325 (13.4)	1487 (15.1)	4.7%
Diabetes with complications	1165 (11.8)	1342 (13.6)	5.4%
Anemia	1329 (13.5)	1619 (16.4)	8.3%
Peripheral vascular disease	1456 (14.8)	1628 (16.5)	4.8%
Cerebrovascular disease	1522 (15.4)	1680 (17.0)	4.3%
Asthma	885 (9.0)	787 (8.0)	3.6%
Depression	812 (8.2)	835 (8.5)	0.8%
Anxiety	722 (7.3)	649 (6.6)	2.9%
Renal disease	1141 (11.6)	1410 (14.3)	8.1%
Myocardial infarction	932 (9.5)	1041 (10.6)	3.7%

Baseline procedures, n (%)			
Gastric bypass surgery	8 (0.1)	9 (0.1)	0.3%
Catheter ablation	415 (4.2)	360 (3.7)	2.9%
Coronary bypass graft	211 (2.1)	302 (3.1)	5.8%
Percutaneous coronary intervention	299 (3.0)	323 (3.3)	1.4%
Cardioversion	174 (1.8)	142 (1.4)	2.6%
Baseline medication use, n (%)			
Nonoral anticoagulants	953 (9.7)	1018 (10.3)	2.2%
Antihyperlipidemics	883 (9.0)	959 (9.7)	2.6%
Antihypertensives	8967 (91.0)	8960 (90.9)	0.2%
Antiplatelet agents	1258 (12.8)	1243 (12.6)	0.5%
Antiarrhythmia agents	8216 (83.3)	8222 (83.4)	0.2%
Beta-blockers	6697 (67.9)	6723 (68.2)	0.6%
Calcium channel blockers	3450 (35.0)	3467 (35.2)	0.4%
Digoxin	897 (9.1)	1061 (10.8)	5.6%
Baseline all-cause HRU counts, median (IQR)			
Inpatient hospitalization	1 (0-1)	1 (0-1)	3.2%
ER visit	0 (0-0)	0 (0-0)	0.4%
Office visit	9 (5-14)	9 (5-15)	4.4%
Outpatient visit	35 (18-64)	39 (20-67)	5.2%
Pharmacy fill	30 (18-46)	32 (20-48)	4.1%
SNF/long-term care (yes), n (%)	418 (4.2)	530 (5.4)	5.3%

Baseline costs ($), median (IQR)			
Inpatient hospitalization	746.85 (0-16,629)	629.04 (0-20,050)	6.4%
ER visit	0 (0-0)	0 (0-0)	0.6%
Office visit	1009.11 (579-1637)	1013.90 (563-1669)	2.1%
Outpatient visit	3323.35 (1194-8779)	3333.69 (1160-8898)	3.8%
Pharmacy fill	2398.28 (790-5303)	2343.03 (769-5270)	2.9%
SNF visit	0 (0-0)	0 (0-0)	3.4%

BMI body mass index, *ER* emergency room, *HRU* healthcare resource utilization, *QCI* Quan-Charlson comorbidity index, *SD* standard deviation, *SNF* skilled nursing facility.

*A standardized mean difference <10% was considered a negligible imbalance.
[†]10% or more in either treatment cohort before matching.

Table S5. Demographic and baseline characteristics after matching for patients with BMI of 35–39 kg/m^2.

	After matching		
**Characteristic**	**Rivaroxaban (n=2643)**	**Warfarin (n=2643)**	**Standardized mean difference***
Age, years, median (IQR)	64 (59-72)	64 (59-72)	0.9%
Sex, n (%)			
Male	1780 (67.3)	1780 (67.3)	0.0%
Female	863 (32.7)	863 (32.7)	0.0%
Insurance type, n (%)			
Commercial	1473 (55.7)	1473 (55.7)	0.0%
Medicare	1170 (44.3)	1170 (44.3)	0.0%
Baseline risk scores, median (IQR)			
QCI	1 (0-2)	1 (0-3)	7.2%
CHA_2DS_2-VASC	3 (2-4)	3 (2-4)	1.7%
HAS-BLED	2 (1-3)	2 (1-3)	2.2%
Baseline concurrent medications, n (%)			
1-4	949 (35.9)	989 (37.4)	3.1%
5-9	1246 (47.1)	1229 (46.5)	1.3%
≥10	448 (17.0)	425 (16.1)	2.3%
Baseline obesity class, n (%)			
Class I (BMI 30-34 kg/m^2)	-	-	-
Class II (BMI 35-39 kg/m^2)	2643 (100)	2643 (100)	-

Class III (BMI ≥40.0 kg/m^2)	-	-	-
Most common baseline comorbidities (>10%[†]), n (%)			
Hypertension	2242 (84.8)	2246 (85.0)	0.4%
Hyperlipidemia	1639 (62.0)	1636 (61.9)	0.2%
Diabetes without chronic complications	1346 (50.9)	1359 (51.4)	1.0%
Congestive heart failure	761 (28.8)	792 (30.0)	2.6%
Osteoarthritis	723 (27.4)	634 (24.0)	7.7%
Chronic pulmonary disease	642 (24.3)	681 (25.8)	3.4%
Cancer	707 (26.8)	708 (26.8)	0.1%
Thyroid disease	470 (17.8)	443 (16.8)	2.7%
Coronary artery disease	368 (13.9)	399 (15.1)	3.3%
Chronic obstructive pulmonary disease	393 (14.9)	426 (16.1)	3.5%
Diabetes with complications	415 (15.7)	455 (17.2)	4.1%
Anemia	351 (13.3)	389 (14.7)	4.1%
Peripheral vascular disease	316 (12.0)	299 (11.3)	2.0%
Cerebrovascular disease	341 (12.9)	364 (13.8)	2.6%
Asthma	241 (9.1)	225 (8.5)	2.1%
Depression	254 (9.6)	249 (9.4)	0.6%
Anxiety	209 (7.9)	175 (6.6)	5.0%
Renal disease	315 (11.9)	394 (14.9)	8.8%
Myocardial infarction	203 (7.7)	216 (8.2)	1.8%

Baseline procedures, n (%)			
Gastric bypass surgery	10 (0.4)	13 (0.5)	1.7%
Catheter ablation	117 (4.4)	103 (3.9)	2.7%
Coronary bypass graft	53 (2.0)	64 (2.4)	2.8%
Percutaneous coronary intervention	61 (2.3)	68 (2.6)	1.7%
Cardioversion	40 (1.5)	35 (1.3)	1.6%
Baseline medication use, n (%)			
Nonoral anticoagulants	284 (10.7)	285 (20.8)	0.1%
Antihyperlipidemics	221 (8.4)	231 (8.7)	1.4%
Antihypertensives	2477 (93.7)	2481 (93.9)	0.6%
Antiplatelet agents	281 (10.6)	274 (10.4)	0.9%
Antiarrhythmia agents	2239 (84.7)	2227 (84.3)	1.3%
Beta-blockers	1830 (69.2)	1829 (69.2)	0.1%
Calcium channel blockers	1024 (38.7)	995 (37.6)	2.3%
Digoxin	256 (9.7)	301 (11.4)	5.5%
Baseline all-cause HRU counts, median (IQR)			
Inpatient hospitalization	1 (0-1)	0 (0-1)	3.7%
ER visit	0 (0-0)	0 (0-0)	1.8%
Office visit	9 (5-14)	9 (5-15)	5.4%
Outpatient visit	36 (19-68)	42 (23-73)	8.0%
Pharmacy fill	36 (22-55)	38 (24-55)	1.4%
SNF/long-term care (yes), n (%)	111 (4.2)	120 (4.5)	1.7%

Baseline costs ($), median (IQR)			
Inpatient hospitalization	213.20 (0-15,722)	0 (0-17,330)	5.2%
ER visit	0 (0-0)	0 (0-0)	1.9%
Office visit	1054.25 (588-1658)	1059.52 (599-1735)	3.1%
Outpatient visit	3461.10 (1216-9090)	3517.45 (1213-9467)	4.1%
Pharmacy fill	3093.07 (1056-6777)	2909.22 (991-6639)	1.5%
SNF visit	0 (0-0)	0 (0-0)	4.9%

BMI body mass index, *ER* emergency room, *HRU* healthcare resource utilization, *QCI* Quan-Charlson comorbidity index, *SD* standard deviation, *SNF* skilled nursing facility.

*A standardized mean difference <10% was considered a negligible imbalance.
[†]10% or more in either treatment cohort before matching.

Table S6. Demographic and baseline characteristics after matching for patients with BMI of ≥40 kg/m^2.

	After matching		
**Characteristic**	**Rivaroxaban (n=6849)**	**Warfarin (n=6849)**	**Standardized mean difference***
Age, years, median (IQR)	62 (56-69)	62 (56-69)	0.6%
Sex, n (%)			
Male	3566 (52.1)	3566 (52.1)	0.0%
Female	3283 (47.9)	3283 (47.9)	0.0%
Insurance type, n (%)			
Commercial	4450 (65.0)	4450 (65.0)	0.0%
Medicare	2399 (35.0)	2399 (35.0)	0.0%
Baseline risk scores, median (IQR)			
QCI	2 (0-3)	2 (0-3)	7.0%
CHA_2DS_2-VASC	3 (2-4)	3 (2-4)	6.7%
HAS-BLED	2 (1-3)	2 (1-3)	5.8%
Baseline concurrent medications, n (%)			
1-4	2283 (33.3)	2212 (32.3)	2.2%
5-9	3137 (45.8)	3144 (45.9)	0.2%
≥10	1429 (20.9)	1493 (21.8)	2.3%
Baseline obesity class, n (%)			
Class I (BMI 30-34 kg/m^2)	-	-	-
Class II (BMI 35-39 kg/m^2)	-	-	-

Class III (BMI ≥40.0 kg/m^2)	6849 (100)	6849 (100)	-
Most common baseline comorbidities (>10%[†]), n (%)			
Hypertension	6139 (89.6)	6088 (88.9)	2.4%
Hyperlipidemia	4478 (65.4)	4270 (62.3)	6.3%
Diabetes without chronic complications	3821 (55.8)	3974 (58.0)	4.5%
Congestive heart failure	2781 (40.6)	2989 (43.6)	6.2%
Osteoarthritis	2123 (31.0)	2090 (30.5)	1.0%
Chronic pulmonary disease	2451 (35.8)	2530 (36.9)	2.4%
Cancer	1780 (26.0)	1862 (27.2)	2.7%
Thyroid disease	1636 (23.9)	1535 (22.4)	3.5%
Coronary artery disease	1118 (16.3)	1278 (18.7)	6.2%
Chronic obstructive pulmonary disease	1436 (21.0)	1514 (22.1)	2.8%
Diabetes with complications	1394 (20.4)	1563 (22.8)	6.0%
Anemia	1311 (19.1)	1565 (22.9)	9.1%
Peripheral vascular disease	1094 (16.0)	1242 (18.1)	5.7%
Cerebrovascular disease	934 (13.6)	1055 (15.4)	5.0%
Asthma	1165 (17.0)	1076 (15.7)	3.5%
Depression	931 (13.6)	964 (14.1)	1.4%
Anxiety	823 (12.0)	731 (10.7)	4.2%
Renal disease	1044 (15.2)	1319 (19.3)	10.6%
Myocardial infarction	724 (10.6)	809 (11.8)	3.9%

Baseline procedures, n (%)			
Gastric bypass surgery	59 (0.9)	57 (0.8)	0.3%
Catheter ablation	279 (4.1)	269 (3.9)	0.7%
Coronary bypass graft	148 (2.2)	216 (3.2)	6.2%
Percutaneous coronary intervention	225 (3.3)	231 (3.4)	0.5%
Cardioversion	181 (2.6)	155 (2.3)	2.5%
Baseline medication use, n (%)			
Nonoral anticoagulants	943 (13.8)	987 (14.4)	1.8%
Antihyperlipidemics	693 (10.1)	759 (11.1)	3.1%
Antihypertensives	6412 (93.6)	6424 (93.8)	0.7%
Antiplatelet agents	846 (12.4)	816 (11.9)	1.3%
Antiarrhythmia agents	5733 (83.7)	5754 (84.0)	0.8%
Beta-blockers	4715 (68.8)	4753 (69.4)	1.2%
Calcium channel blockers	2633 (38.4)	2683 (39.2)	1.5%
Digoxin	692 (10.1)	748 (10.9)	2.7%
Baseline all-cause HRU counts, median (IQR)			
Inpatient hospitalization	1 (0-2)	1 (0-2)	3.7%
ER visit	0 (0-1)	0 (0-1)	0.2%
Office visit	10 (6-16)	11 (6-17)	6.0%
Outpatient visit	45 (24-81)	49 (26-86)	6.1%
Pharmacy fill	39 (24-61)	42 (26-63)	5.1%
SNF/long-term care (yes), n (%)	349 (5.1)	457 (6.7)	6.7%

Baseline costs ($), median (IQR)			
Inpatient hospitalization	8870.64 (0-27,455)	10,084.08 (0-33,776)	8.8%
ER visit	0 (0-411)	0 (0-491)	0.5%
Office visit	1189.22 (696-1933)	1222.20 (693-2024)	2.7%
Outpatient visit	4418.61 (1595-11,525)	4653.24 (1699-12,216)	4.1%
Pharmacy fill	3171.01 (1060-7160)	3324.75 (1098-7585)	4.9%
SNF visit	0 (0-0)	0 (0-0)	3.0%

BMI body mass index, *ER* emergency room, *HRU* healthcare resource utilization, *QCI* Quan-Charlson comorbidity index, *SD* standard deviation, *SNF* skilled nursing facility.

*A standardized mean difference <10% was considered a negligible imbalance.
[†]10% or more in either treatment cohort before matching.

Table S7. Demographic and baseline characteristics after matching for patients with an index year of 2014 and after.

	After matching		
**Characteristic**	**Rivaroxaban (n=10,133)**	**Warfarin (n=10,133)**	**Standardized mean difference***
Age, years, median (IQR)	65 (59-74)	65 (59-74)	0.5%
Sex, n (%)			
Male	6245 (61.6)	6245 (61.6)	0.0%
Female	3888 (38.4)	3888 (38.4)	0.0%
Insurance type, n (%)			
Commercial	5209 (51.4)	5209 (51.4)	0.0%
Medicare	4924 (48.6)	4924 (48.6)	0.0%
Baseline risk scores, median (IQR)			
QCI	2 (0-3)	2 (0-3)	3.9%
CHA_2DS_2-VASC	3 (2-5)	3 (2-5)	2.4%
HAS-BLED	2 (2-3)	2 (2-3)	2.7%
Baseline concurrent medications, n (%)			
1-4	4060 (40.1)	4086 (40.3)	0.5%
5-9	4467 (44.1)	4416 (43.6)	1.0%
≥10	1606 (15.8)	1631 (16.1)	0.7%
Baseline obesity class, n (%)			
Class I (BMI 30-34 kg/m^2)	4896 (48.3)	4947 (48.8)	1.0%
Class II (BMI 35-39 kg/m^2)	1391 (13.7)	1376 (13.6)	0.4%

Class III (BMI ≥40.0 kg/m^2)	3846 (38.0)	3810 (37.6)	0.7%
Most common baseline comorbidities (>10%[†]), n (%)			
Hypertension	9048 (89.3)	9058 (89.4)	0.3%
Hyperlipidemia	6951 (68.6)	6802 (67.1)	3.1%
Diabetes without chronic complications	4861 (48.0)	4905 (48.4)	0.9%
Congestive heart failure	3735 (36.9)	3867 (38.2)	2.7%
Osteoarthritis	2897 (28.6)	2927 (28.9)	0.7%
Chronic pulmonary disease	3142 (31.0)	3271 (32.3)	2.7%
Cancer	2671 (26.4)	2677 (26.4)	0.1%
Thyroid disease	2248 (22.2)	2181 (21.5)	1.6%
Coronary artery disease	2233 (22.0)	2452 (24.2)	5.1%
Chronic obstructive pulmonary disease	1913 (18.9)	2058 (20.3)	3.6%
Diabetes with complications	1999 (19.7)	2078 (20.5)	1.9%
Anemia	2134 (21.1)	2343 (23.1)	5.0%
Peripheral vascular disease	1902 (18.8)	2010 (19.8)	2.7%
Cerebrovascular disease	1697 (16.7)	1737 (17.1)	1.1%
Asthma	1350 (13.3)	1247 (12.3)	3.0%
Depression	1250 (12.3)	1294 (12.8)	1.3%
Anxiety	1129 (11.1)	1058 (10.4)	2.3%
Renal disease	1758 (17.3)	2019 (19.9)	6.6%
Myocardial infarction	1284 (12.7)	1372 (13.5)	2.6%

Baseline procedures, n (%)			
Gastric bypass surgery	44 (0.4)	48 (0.5)	0.6%
Catheter ablation	259 (2.6)	292 (2.9)	2.0%
Coronary bypass graft	326 (3.2)	395 (3.9)	3.7%
Percutaneous coronary intervention	405 (4.0)	417 (4.1)	0.6%
Cardioversion	156 (1.5)	162 (1.6)	0.5%
Baseline medication use, n (%)			
Nonoral anticoagulants	1295 (12.8)	1355 (13.4)	1.8%
Antihyperlipidemics	984 (9.7)	1065 (10.5)	2.7%
Antihypertensives	9271 (91.5)	9249 (91.3)	0.8%
Antiplatelet agents	1354 (13.4)	1354 (13.4)	0.0%
Antiarrhythmia agents	8085 (79.8)	8158 (80.5)	1.8%
Beta-blockers	6576 (64.9)	6627 (65.4)	1.1%
Calcium channel blockers	3614 (35.7)	3712 (36.6)	2.0%
Digoxin	656 (6.5)	638 (6.3)	0.7%
Baseline all-cause HRU counts, median (IQR)			
Inpatient hospitalization	1 (0-2)	1 (0-2)	3.5%
ER visit	0 (0-1)	0 (0-1)	1.6%
Office visit	9 (5-15)	9 (5-15)	1.7%
Outpatient visit	39 (19-73)	41 (20-74)	2.4%
Pharmacy fill	32 (19-51)	33 (19-52)	1.7%
SNF/long-term care (yes), n (%)	636 (6.3)	764 (7.5)	5.0%

Baseline costs ($), median (IQR)			
Inpatient hospitalization	8947.96 (0-32,616)	10,027.97 (0-36,809)	6.4%
ER visit	0 (0-51)	0 (0-152)	0.7%
Office visit	1056.73 (580-1775)	1065.26 (584-1796)	1.0%
Outpatient visit	3690.08 (1260-10,338)	3826.07 (1327-10,626)	4.5%
Pharmacy fill	2016.10 (595-5636)	2071.36 (614-5801)	3.1%
SNF visit	0 (0-0)	0 (0-0)	2.4%

BMI body mass index, *ER* emergency room, *HRU* healthcare resource utilization, *QCI* Quan-Charlson comorbidity index, *SD* standard deviation, *SNF* skilled nursing facility.

*A standardized mean difference <10% was considered a negligible imbalance.
[†]10% or more in either treatment cohort before matching.

Table S8. Demographic and baseline characteristics after matching for patients with a baseline obesity diagnosis.

	After matching		
**Characteristic**	**Rivaroxaban (n=6331)**	**Warfarin (n=6331)**	**Standardized mean difference***
Age, years, median (IQR)	63 (58-72)	64 (58-72)	0.7%
Sex, n (%)			
Male	3748 (59.2)	3748 (59.2)	0.0%
Female	2583 (40.8)	2583 (40.8)	0.0%
Insurance type, n (%)			
Commercial	3564 (56.3)	3564 (56.3)	0.0%
Medicare	2767 (43.7)	2767 (43.7)	0.0%
Baseline risk scores, median (IQR)			
QCI	2 (0-3)	2 (0-3)	5.7%
CHA_2DS_2-VASC	3 (2-4)	3 (2-5)	5.6%
HAS-BLED	2 (1-3)	2 (2-3)	4.8%
Baseline concurrent medications, n (%)			
1-4	2452 (38.7)	2384 (37.7)	2.2%
5-9	2740 (43.3)	2775 (43.8)	1.1%
≥10	1139 (18.0)	1172 (18.5)	1.3%
Baseline obesity class, n (%)			
Class I (BMI 30-34 kg/m^2)	-	-	-
Class II (BMI 35-39 kg/m^2)	-	-	-

Class III (BMI ≥40.0 kg/m^2)	-	-	-
Most common baseline comorbidities (>10%†), n (%)			
Hypertension	5672 (89.6)	5739 (90.6)	3.5%
Hyperlipidemia	4542 (71.7)	4424 (69.9)	4.1%
Diabetes without chronic complications	3128 (49.4)	3218 (50.8)	2.8%
Congestive heart failure	2450 (38.7)	2634 (41.6)	5.9%
Osteoarthritis	1976 (31.2)	1919 (30.3)	2.0%
Chronic pulmonary disease	2175 (34.4)	2285 (36.1)	3.6%
Cancer	1748 (27.6)	1767 (27.9)	0.7%
Thyroid disease	1510 (23.9)	1406 (22.2)	3.9%
Coronary artery disease	1285 (20.3)	1445 (22.9)	6.1%
Chronic obstructive pulmonary disease	1341 (21.2)	1446 (22.8)	4.0%
Diabetes with complications	1273 (20.1)	1356 (21.4)	3.2%
Anemia	1265 (20.0)	1647 (26.0)	14.4%
Peripheral vascular disease	1165 (18.4)	1279 (20.2)	4.6%
Cerebrovascular disease	990 (15.6)	1066 (16.8)	3.3%
Asthma	924 (14.6)	903 (14.3)	0.9%
Depression	900 (14.2)	967 (15.3)	3.0%
Anxiety	859 (13.6)	810 (12.8)	2.3%
Renal disease	1096 (17.3)	1299 (20.5)	8.2%
Myocardial infarction	774 (12.2)	828 (13.1)	2.6%

Baseline procedures, n (%)			
Gastric bypass surgery	70 (1.1)	77 (1.2)	1.0%
Catheter ablation	271 (4.3)	266 (4.2)	0.4%
Coronary bypass graft	184 (2.9)	259 (4.1)	6.5%
Percutaneous coronary intervention	220 (3.5)	240 (3.8)	1.7%
Cardioversion	154 (2.4)	133 (2.1)	2.2%
Baseline medication use, n (%)			
Nonoral anticoagulants	971 (15.3)	1007 (15.9)	1.6%
Antihyperlipidemics	658 (10.4)	726 (11.5)	3.4%
Antihypertensives	5784 (91.4)	5826 (92.0)	2.4%
Antiplatelet agents	814 (12.9)	819 (12.9)	0.2%
Antiarrhythmia agents	5179 (81.8)	5242 (82.8)	2.6%
Beta-blockers	4181 (66.0)	4276 (67.5)	3.2%
Calcium channel blockers	2400 (37.9)	2459 (38.8)	1.9%
Digoxin	484 (7.6)	515 (8.1)	1.8%
Baseline all-cause HRU counts, median (IQR)			
Inpatient hospitalization	1 (0-2)	1 (0-2)	2.4%
ER visit	0 (0-1)	0 (0-1)	2.7%
Office visit	10 (6-16)	10 (6-17)	4.4%
Outpatient visit	47 (25-84)	49 (27-84)	3.3%
Pharmacy fill	36 (21-56)	38 (23-58)	4.0%
SNF/long-term care (yes), n (%)	389 (6.1)	467 (7.4)	4.9%

Baseline costs ($), median (IQR)			
Inpatient hospitalization	10,529.54 (0-31,370)	11,910.90 (0-37,032)	5.1%
ER visit	0 (0-412)	0 (0-488)	0.3%
Office visit	1203.85 (694-1922)	1215.03 (696-1984)	3.0%
Outpatient visit	4648.44 (1714-11,781)	4800.46 (1771-11,825)	4.5%
Pharmacy fill	2686.71 (841-6502)	2730.59 (875-6567)	1.2%
SNF visit	0 (0-0)	0 (0-0)	1.1%

BMI body mass index, *ER* emergency room, *HRU* healthcare resource utilization, *QCI* Quan-Charlson comorbidity index, *SD* standard deviation, *SNF* skilled nursing facility.

*A standardized mean difference <10% was considered a negligible imbalance.
[†]10% or more in either treatment cohort before matching.

Table S9. Sensitivity analyses for all-cause HRU for an index year of 2014 and after, for obesity based on baseline obesity diagnosis, and for on-treatment approach.

Rate (SD), PPPY	Rivaroxaban	Warfarin	Adjusted difference (95% CI)	*P* value
**Index year of 2014 and after (n=10,133 in each arm)**				
Hospital admission	1.29 (3.19)	1.50 (3.70)	0.86 (0.84, 0.88)	<0.0001
Length of stay in hospitalized patients (days)	15.26 (34.09)	17.40 (34.74)	–2.14 (–2.87, –1.37)	<0.0001
Length of stay in all patients (days)	8.46 (26.49)	9.81 (27.47)	–1.35 (–2.28, –0.30)	0.0129
ER visit	0.53 (2.08)	0.63 (2.74)	0.84 (0.81, 0.88)	<0.0001
Outpatient visit	79.63 (90.52)	102.14 (123.52)	0.78 (0.78, 0.78)	<0.0001
Physician office visit	14.76 (12.61)	18.48 (17.04)	0.80 (0.79, 0.80)	<0.0001
**Obesity based on baseline obesity diagnosis (n=6331 in each arm)**				
Hospital admission	1.22 (3.05)	1.44 (3.50)	0.84 (0.82, 0.87)	<0.0001
Length of stay in hospitalized patients (days)	13.51 (29.99)	16.10 (28.40)	–2.59 (–3.38, –1.75)	<0.0001
Length of stay in all patients (days)	7.50 (23.33)	9.03 (22.73)	–1.53 (–2.55, –0.35)	0.0124
ER visit	0.53 (1.44)	0.61 (2.25)	0.87 (0.83, 0.91)	<0.0001
Outpatient visit	80.05 (91.46)	98.57 (111.13)	0.81 (0.81, 0.82)	<0.0001
Physician office visit cost	15.21 (13.65)	17.82 (16.22)	0.85 (0.85, 0.86)	<0.0001
**On-treatment approach (n=19,990 in each arm)**				

Hospital admission	1.56 (4.57)	1.66 (5.41)	0.94 (0.92, 0.95)	<0.0001
Length of stay in hospitalized patients (days)	21.95 (60.92)	28.92 (100.28)	–6.97 (–7.98, –5.92)	<0.0001
Length of stay in all patients (days)	8.37 (39.11)	10.38 (61.67)	–2.01 (–2.81, –1.13)	<0.0001
ER visit	0.63 (3.78)	0.67 (2.94)	0.93, (0.91, 0.96)	<0.0001
Outpatient visit	83.27 (132.05)	107.18 (167.16)	0.78 (0.78, 0.78)	<0.0001
Physician office visit	17.39 (25.83)	20.95 (29.72)	0.83 (0.83, 0.83)	<0.0001

CI confidence interval, *ER* emergency room, *NVAF* nonvalvular atrial fibrillation, *PPPY* per patient per year, *SD* standard deviation, *SNF* skilled nursing facility.

Table S10. Sensitivity analyses for all-cause costs for an index year of 2014 and after, for obesity based on baseline obesity diagnosis, and for on-treatment approach.

Mean cost (SD), PPPY	Rivaroxaban	Warfarin	Adjusted difference (95% CI)	*P* value
**Index year of 2014 and after (n=10,133 in each arm)**				
Total medical cost	50,903 (179,529)	53,258 (129,228)	–2355 (–4257, –378)	0.0200
Inpatient visit cost	28,123 (163,269)	27,655 (102,028)	469 (–1495, 2580)	0.6492
ER cost	1083 (5649)	1080 (5592)	3 (–68, 80)	0.9307
Outpatient visit cost	19,532 (65,736)	21,959 (65,845)	–2428 (–3176, –1650)	<0.0001
Physician office visit cost	1885 (2772)	2137 (2717)	–252 (–305, –198)	<0.0001
SNF cost	283 (2256)	430 (4533)	–147 (–167, –125)	<0.0001
Outpatient pharmacy cost	10,301 (16,401)	7375 (15,893)	2926 (2629, 3231)	<0.0001
Total healthcare cost	61,204 (181,382)	60,633 (131,541)	571 (–1372, 2578)	0.5691
**Obesity based on baseline obesity diagnosis (n=6331 in each arm)**				
Total medical cost	35,100 (83,578)	41,353 (122,744)	–6253 (–7849, –4581)	<0.0001
Inpatient visit cost	17,682 (71,317)	21,249 (105,281)	–3567 (–5068, –1927)	<0.0001
ER cost	902 (3364)	940 (3680)	–38 (–111, 41)	0.3335
Outpatient visit cost	14,368 (34,256)	16,749 (49,069)	–2381 (–3051, –1680)	<0.0001
Physician office visit cost	1826 (2993)	1996 (2507)	–170 (–236, –101)	<0.0001
SNF cost	326 (2497)	419 (2699)	–94 (–123, –62)	<0.0001
Outpatient pharmacy cost	10,252 (16,432)	6993 (13,823)	3259 (2812, 3726)	<0.0001
Total cost	45,352 (86,978)	48,346 (124,392)	–2994 (–4668, –1257)	0.0009

On-treatment approach (n=19,990 in each arm)				
Total medical cost	45,472 (178,688)	48,949 (214,535)	–3478 (–4944, –1963)	<0.0001
Inpatient visit cost	24,372 (168,522)	25,457 (183,105)	–1084 (–2517, 438)	0.1592
ER cost	1103 (7337)	1138 (6491)	–35 (–95, 28)	0.2644
Outpatient visit cost	17,628 (49,825)	19,638 (101,191)	–2010 (–2591, –1409)	<0.0001
Physician office visit cost	2163 (3528)	2401 (4058)	–238 (–294, –179)	<0.0001
SNF cost	207 (2549)	318 (4440)	–110 (–122, –99)	<0.0001
Outpatient pharmacy cost	15,293 (47,245)	7495 (19,927)	7798 (7462, 8141)	<0.0001
Total cost	60,764 (185,161)	56,444 (216,685)	4320 (2802, 5878)	<0.0001

CI confidence interval, *ER* emergency room, *NVAF* nonvalvular atrial fibrillation, *PPPY* per patient per year, *SD* standard deviation, *SNF* skilled nursing facility.

Table S11. NVAF-related HRU overall and for subgroups of patients by number of concurrent medications and obesity category and for sensitivity analyses of an index year of 2014 and after, for obesity based on baseline obesity diagnosis, and for on-treatment approach.

Rate (SD), PPPY	Rivaroxaban	Warfarin	Adjusted difference (95% CI)	*P* value
**Overall (N=19,990 in each arm)**				
Hospital admission	0.73 (2.09)	0.78 (2.23)	0.93 (0.91, 0.96)	<0.0001
Length of stay in hospitalized patients (days)	11.65 (27.08)	14.77 (32.48)	–3.12 (–3.56, –2.65)	<0.0001
Length of stay in all patients (days)	5.38 (19.30)	6.44 (22.67)	–1.06 (–1.53, –0.55)	0.0001
ER visit	0.17 (1.12)	0.18 (1.37)	0.94 (0.90, 0.99)	0.0139
Outpatient visit	16.19 (43.35)	21.12 (43.08)	0.77 (0.76, 0.77)	<0.0001
Physician office visit	4.38 (7.59)	5.61 (9.44)	0.78 (0.77, 0.79)	<0.0001
**Concurrent medications on index date: 1-4 (n=7593 in each arm)**				
Hospital admission	0.57 (1.98)	0.57 (1.86)	1.00 (0.96, 1.04)	0.9692
Length of stay in hospitalized patients (days)	8.27 (21.33)	10.47 (25.33)	–2.20 (–2.74, –1.62)	<0.0001
Length of stay in all patients (days)	3.42 (14.30)	3.91 (16.28)	–0.49 (–0.98, 0.07)	0.0843
ER visit	0.16 (1.15)	0.13 (0.71)	1.21 (1.11, 1.31)	<0.0001
Outpatient visit	16.90 (52.01)	20.83 (35.46)	0.81 (0.81, 0.82)	<0.0001
Physician office visit	4.42 (7.89)	5.37 (10.02)	0.82 (0.81, 0.84)	<0.0001
**Concurrent medications on index date: 5-9 (n=8626 in each arm)**				

Hospital admission	0.73 (2.07)	0.78 (2.31)	0.93 (0.90, 0.97)	<0.0001
Length of stay in hospitalized patients (days)	11.46 (24.18)	13.93 (32.16)	–2.47 (–3.10, –1.80)	<0.0001
Length of stay in all patients (days)	5.47 (17.65)	6.29 (22.70)	–0.82 (–1.52, –0.02)	0.0440
ER visit	0.19 (1.81)	0.18 (1.24)	1.03 (0.96, 1.10)	0.4104
Outpatient visit	15.66 (34.21)	20.50 (32.46)	0.76 (0.76, 0.77)	<0.0001
Physician office visit	4.40 (7.06)	5.80 (9.39)	0.76 (0.75, 0.77)	<0.0001
**Concurrent medications on index date: ≥10 (n=2992 in each arm)**				
Hospital admission	1.12 (2.41)	1.24 (2.80)	0.91 (0.87, 0.95)	<0.0001
Length of stay in hospitalized patients (days)	18.46 (39.38)	20.63 (36.53)	–2.18 (–3.71, –0.50)	0.0118
Length of stay in all patients (days)	10.28 (30.83)	11.39 (29.01)	–1.10 (–3.09, 1.37)	0.3563
ER visit	0.19 (0.79)	0.23 (2.46)	0.82 (0.73, 0.92)	0.0005
Outpatient visit	14.55 (39.37)	23.28 (76.73)	0.63 (0.62, 0.63)	<0.0001
Physician office visit	3.92 (5.40)	6.11 (9.87)	0.64 (0.63, 0.66)	<0.0001
**BMI: 30-34 kg/m^2 (n=9858 in each arm)**				
Hospital admission	0.67 (1.90)	0.71 (2.01)	0.95 (0.92, 0.98)	0.0043
Length of stay in hospitalized patients (days)	10.62 (26.39)	12.55 (26.17)	–1.92 (–2.50, –1.31)	<0.0001
Length of stay in all patients (days)	4.81 (18.53)	5.30 (18.11)	–0.49 (–1.08, 0.18)	0.1414

ER visit	0.15 (1.00)	0.16 (1.12)	0.97 (0.90, 1.04)	0.4054
Outpatient visit	15.93 (40.85)	19.40 (32.12)	0.82 (0.82, 0.83)	<0.0001
Physician office visit	4.41 (8.05)	5.60 (9.62)	0.79 (0.78, 0.80)	<0.0001
**BMI: 35-39 kg/m^2 (n=2643 in each arm)**				
Hospital admission	0.66 (2.12)	0.70 (1.94)	0.95 (0.89, 1.02)	0.1579
Length of stay in hospitalized patients (days)	11.52 (26.42)	13.59 (29.33)	–2.07 (–3.26, –0.74)	0.0030
Length of stay in all patients (days)	5.26 (18.74)	5.82 (20.33)	–0.56 (–1.72, 0.94)	0.4304
ER visit	0.15 (1.50)	0.14 (0.67)	1.07 (0.93, 1.23)	0.3469
Outpatient visit	16.32 (61.54)	20.40 (30.50)	0.80 (0.79, 0.81)	<0.0001
Physician office visit	4.08 (5.58)	5.20 (7.52)	0.79 (0.77, 0.81)	<0.0001
**BMI: ≥40 kg/m^2 (n=6849 in each arm)**				
Hospital admission	0.81 (2.10)	0.89 (2.22)	0.91 (0.87, 0.94)	<0.0001
Length of stay in hospitalized patients (days)	12.88 (28.35)	17.58 (42.40)	–4.70 (–5.50, –3.84)	<0.0001
Length of stay in all patients (days)	6.17 (20.68)	8.08 (30.06)	–1.91 (–2.78, –0.89)	0.0006
ER visit	0.19 (1.08)	0.21 (1.82)	0.89 (0.83, 0.96)	0.0033
Outpatient visit	16.40 (37.66)	23.49 (53.17)	0.70 (0.69, 0.70)	<0.0001
Physician office visit	4.31 (5.88)	5.78 (8.95)	0.75 (0.74, 0.76)	<0.0001
**Index year of 2014 and after (n=10,133 in each arm)**				

Hospital admission	0.83 (2.31)	0.93 (2.42)	0.89 (0.86, 0.91)	<0.0001
Length of stay in hospitalized patients (days)	13.81 (31.95)	15.99 (31.95)	–2.18 (–2.91, –1.42)	<0.0001
Length of stay in all patients (days)	6.41 (22.82)	7.40 (23.15)	–0.99 (–175, –0.13)	0.0256
ER visit	0.19 (1.63)	0.21 (1.75)	0.92 (0.86, 0.97)	0.0047
Outpatient visit	16.70 (33.92)	26.15 (50.53)	0.64 (0.63, 0.64)	<0.0001
Physician office visit	4.54 (6.61)	7.03 (11.05)	0.65 (0.64, 0.65)	<0.0001
**Obesity based on baseline obesity diagnosis (n=6331 in each arm)**				
Hospital admission	0.67 (1.67)	0.80 (2.01)	0.84 (0.81, 0.87)	<0.0001
Length of stay in hospitalized patients (days)	11.48 (24.77)	14.33 (25.95)	–2.85 (–3.58, –2.08)	<0.0001
Length of stay in all patients (days)	5.30 (17.78)	6.53 (18.92)	–1.23 (–2.01, –0.32)	0.0097
ER visit	0.17 (0.69)	0.21 (1.09)	0.82 (0.76, 0.89)	<0.0001
Outpatient visit	13.93 (27.85)	21.73 (31.75)	0.64 (0.64, 0.65)	<0.0001
Physician office visit	4.33 (6.65)	6.06 (9.95)	0.72 (0.70, 0.73)	<0.0001
**On-treatment approach (n=19,990 in each arm)**				
Hospital admission	0.88 (2.93)	0.94 (4.12)	0.95 (0.93, 0.97)	<0.0001
Length of stay in hospitalized patients (days)	19.26 (51.91)	28.36 (107.31)	–9.10 (–10.11, –8.04)	<0.0001
Length of stay in all patients (days)	5.81 (29.84)	7.52 (56.66)	–1.71 (–2.30, –1.07)	<0.0001

ER visit	0.26 (3.31)	0.24 (1.99)	1.06 (1.02, 1.10)	0.0069
Outpatient visit	21.67 (72.07)	30.11 (67.09)	0.72 (0.72, 0.72)	<0.0001
Physician office visit	6.65 (19.46)	8.50 (20.79)	0.78 (0.78, 0.79)	<0.0001

BMI body mass index, *CI* confidence interval, *ER* emergency room, *HRU* healthcare resource utilization, *NVAF* nonvalvular atrial fibrillation, *PPPY* per patient per year, *SD* standard deviation, *SNF* skilled nursing facility.

A medical service claim was considered NVAF-related if it was associated with a diagnosis of NVAF in the first or second diagnosis position.

Table S12. NVAF-related medical costs (2020 US dollars) overall and for subgroups of patients by number of concurrent medications and obesity category, and for sensitivity analyses of index year of 2014 and after, for obesity based on baseline obesity diagnosis, and for on-treatment approach.

Mean cost (SD), PPPY	Rivaroxaban	Warfarin	Adjusted difference (95% CI)	*P* value
**Overall (N=19,990 in each arm)**				
Total medical cost	24,518 (122,134)	24,374 (105,248)	144 (–756, 1079)	0.7570
Inpatient visit cost	17,224 (118,014)	18,545 (98,580)	–1321 (–2234, –357)	0.0078
ER cost	374 (5204)	309 (3235)	65 (47, 85)	<0.0001
Outpatient visit cost	6283 (27,803)	4835 (33,635)	1449 (1197, 1711)	<0.0001
Physician office visit cost	572 (1238)	597 (1061)	–25 (–42, –8)	0.0043
SNF cost	67 (1614)	90 (1334)	–23 (–27, –20)	<0.0001
**Concurrent medications on index date: 1-4 (n=7593 in each arm)**				
Total medical cost	20,047 (84,727)	20,095 (90,417)	–48 (–1233, 1212)	0.9387
Inpatient visit cost	12,441 (79,113)	13,990 (79,127)	–1549 (–2628, –368)	0.0112
ER cost	412 (4344)	245 (1398)	167 (134, 203)	<0.0001
Outpatient visit cost	6560 (28,829)	5213 (32,836)	1348 (927, 1797)	<0.0001
Physician office visit cost	587 (1076)	601 (1178)	–13 (–41, 16)	0.3660
SNF cost	47 (951)	48 (991)	–1 (–4, 3)	0.5751
**Concurrent medications on index date: 5-9 (n=8626 in each arm)**				
Total medical cost	23,697 (83,635)	23,151 (93,876)	545 (–742, 1907)	0.4138
Inpatient visit cost	16,609 (78,151)	17,591 (90,209)	–982 (–2284, 431)	0.1679

ER cost	460 (7481)	317 (3182)	143 (108, 181)	<0.0001
Outpatient visit cost	5996 (25,962)	4539 (22,623)	1457 (1099, 1837)	<0.0001
Physician office visit cost	558 (917)	604 (1053)	–46 (–70, –20)	0.0005
SNF cost	75 (2217)	102 (1707)	–27 (–32, –21)	<0.0001
**Concurrent medications on index date: ≥10 (n=2992 in each arm)**				
Total medical cost	38,670 (240,136)	35,165 (96,732)	3505 (–79, 7455)	0.0555
Inpatient visit cost	32,222 (238,514)	29,348 (90,363)	2874 (–1131, 7447)	0.1677
ER cost	358 (1667)	403 (5808)	–45 (–90, 6)	0.0817
Outpatient visit cost	5508 (24,852)	4662 (26,962)	846 (283, 1473)	0.0025
Physician office visit cost	497 (781)	634 (1118)	–138 (–176, –96)	<0.0001
SNF cost	87 (866)	119 (1005)	–32 (–42, –20)	<0.0001
**BMI: 30-34 kg/m^2 (n=9858 in each arm)**				
Total medical cost	22,999 (138,389)	20,611 (71,874)	2388 (1210, 3630)	<0.0001
Inpatient visit cost	15,828 (135,572)	15,142 (66,768)	687 (–497, 1965)	0.2632
ER cost	372 (6402)	282 (3052)	91 (64, 119)	<0.0001
Outpatient visit cost	6161 (24,752)	4522 (23,836)	1639 (1293, 2066)	<0.0001
Physician office visit cost	569 (1076)	587 (1033)	–17 (–41, 7)	0.1595
SNF cost	69 (2100)	81 (1390)	–11 (–16, –6)	<0.0001
**BMI: 35-39 kg/m^2 (n=2643 in each arm)**				
Total medical cost	21,846 (68,328)	21,543 (71,650)	303 (–1813, 2648)	0.7879
Inpatient visit cost	14,300 (54,712)	16,152 (65,883)	–1852 (–3832, 448)	0.1095

ER cost	346 (5859)	244 (1230)	102 (57, 153)	<0.0001
Outpatient visit cost	6625 (39,554)	4482 (24,849)	2143 (1438, 2932)	<0.0001
Physician office visit cost	521 (837)	558 (1050)	–37 (–78, 7)	0.1007
SNF cost	55 (672)	108 (1338)	–52 (–59, –45)	<0.0001
**BMI ≥40 kg/m^2 (n=6849 in each arm)**				
Total medical cost	28,304 (111,019)	30,588 (195,217)	–2285 (–4071, –378)	0.0196
Inpatient visit cost	21,067 (107,620)	24,747 (193,267)	–3680 (–5544, –1634)	0.0007
ER cost	385 (2135)	394 (4154)	–9 (–41, 27)	0.6128
Outpatient visit cost	6238 (25,212)	4701 (21,982)	1536 (1114, 1989)	<0.0001
Physician office visit cost	558 (799)	627 (1036)	–69 (–97, –39)	<0.0001
SNF cost	57 (957)	121 (1544)	–64 (–68, –59)	<0.0001
**Index year of 2014 and after (n=10,133 in each arm)**				
Total medical cost	30,143 (159,627)	28,689 (97,848)	1454 (–54, 3041)	0.0591
Inpatient visit cost	22,235 (156,723)	21,451 (91,801)	784 (–852, 2551)	0.3572
ER cost	435 (4460)	390 (4316)	44 (14, 77)	0.0040
Outpatient visit cost	6810 (27,316)	5969 (29,850)	841 (464, 1239)	<0.0001
Physician office visit cost	608 (1118)	758 (1258)	–150 (–174, –124)	<0.0001
SNF cost	57 (723)	123 (1767)	–66 (–70, –62)	<0.0001
**Obesity based on baseline obesity diagnosis (n=6331 in each arm)**				
Total medical cost	8424 (12,198)	8433 (12,030)	–9 (–477, 486)	0.9701
Inpatient visit cost	4426 (9228)	4430 (9067)	–4 (–379, 404)	0.9820

ER cost	269 (1469)	284 (1669)	–15 (–38, 10)	0.2188
Outpatient visit cost	3130 (7059)	2991 (6798)	139 (–65, 357)	0.1867
Physician office visit cost	541 (1072)	636 (1096)	–95 (–122, –65)	<0.0001
SNF cost	60 (766)	93 (867)	–33 (–38, –28)	<0.0001
**On-treatment approach (n=19,990 in each arm)**				
Total medical cost	13,995 (57,055)	11,972 (50,568)	2023 (1465, 2604)	<0.0001
Inpatient visit cost	6609 (49,408)	5599 (38,412)	1010 (628, 1416)	<0.0001
ER cost	416 (5770)	381 (4210)	34 (12, 58)	0.0025
Outpatient visit cost	6077 (25,293)	5006 (29,223)	1071 (798, 1356)	<0.0001
Physician office visit cost	855 (2399)	927 (2382)	–72 (–103, –40)	<0.0001
SNF cost	40 (833)	60 (1171)	–20 (–22, –18)	<0.0001

BMI body mass index, *CI* confidence interval, *ER* emergency room, *NVAF* nonvalvular atrial fibrillation, *PPPY* per patient per year, *SD* standard deviation, *SNF* skilled nursing facility.

A medical service claim was considered NVAF-related if it was associated with a diagnosis of NVAF in the first or second diagnosis position.

Figure S1. Study design.

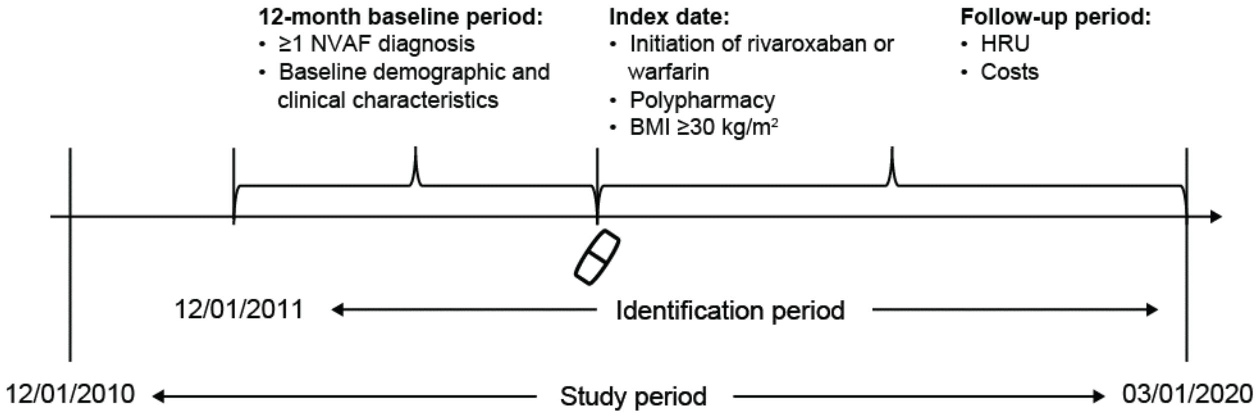

Patients were followed up to the earlier event of continuous benefit enrollment end or study period end on March 1, 2020.

BMI body mass index, *HRU* healthcare resource utilization, *NVAF* nonvalvular atrial fibrillation.

Figure S2. Polypharmacy definition including days of supply and the grace period. In this example, drugs A and B would be counted when determining polypharmacy but drugs C and D would not be counted. See Data S3 for codes of medications of interest.

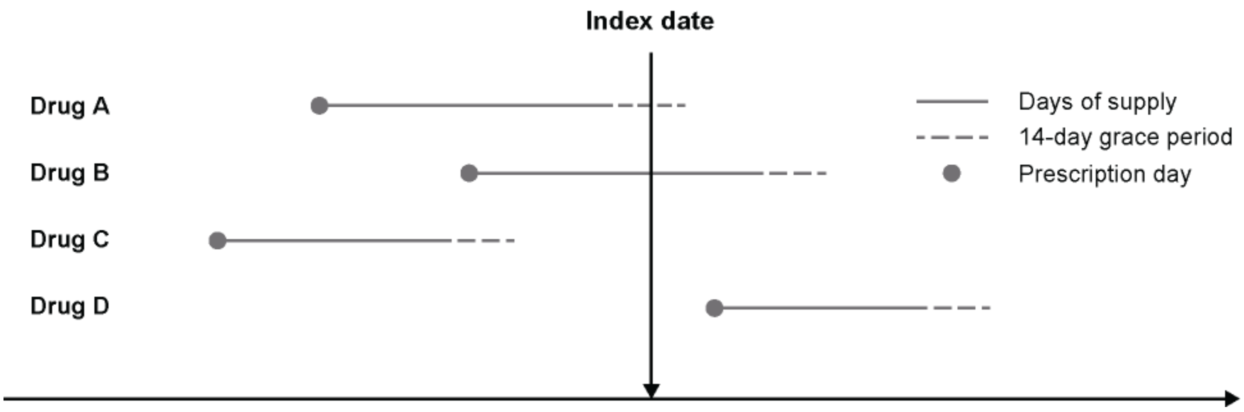

Supplement: Supplementary file 1 — Data S1–S3 Tables S1–S12 Figures S1–S2 [file JAH3-14-e036401-s001.pdf]
